# Locus Coeruleus Activation Patterns Differentially Modulate Odor Discrimination Learning and Odor Valence in Rats

**DOI:** 10.1093/texcom/tgab026

**Published:** 2021-04-05

**Authors:** Abhinaba Ghosh, Faghihe Massaeli, Kyron D Power, Tamunotonye Omoluabi, Sarah E Torraville, Julia B Pritchett, Tayebeh Sepahvand, Vanessa D Strong, Camila Reinhardt, Xihua Chen, Gerard M Martin, Carolyn W Harley, Qi Yuan

**Affiliations:** 1 Biomedical Sciences, Faculty of Medicine, Memorial University of Newfoundland, St. John‘s, NL A1B 3V6, Canada; 2 Psychology Department, Faculty of Science, Memorial University of Newfoundland, St. John’s, NL A1B 3X9, Canada

**Keywords:** locus coeruleus, odor discrimination, odor valence, optogenetic

## Abstract

The locus coeruleus (LC) produces phasic and tonic firing patterns that are theorized to have distinct functional consequences. However, how different firing modes affect learning and valence encoding of sensory information are unknown. Here, we show bilateral optogenetic activation of rat LC neurons using 10-Hz phasic trains of either 300 ms or 10 s accelerated acquisition of a similar odor discrimination. Similar odor discrimination learning was impaired by noradrenergic blockade in the piriform cortex (PC). However, 10-Hz phasic light-mediated learning facilitation was prevented by a dopaminergic antagonist in the PC, or by ventral tegmental area (VTA) silencing with lidocaine, suggesting a LC–VTA–PC dopamine circuitry involvement. Ten-hertz tonic stimulation did not alter odor discrimination acquisition, and was ineffective in activating VTA DA neurons. For valence encoding, tonic stimulation at 25 Hz induced conditioned odor aversion, whereas 10-Hz phasic stimulations produced an odor preference. Both conditionings were prevented by noradrenergic blockade in the basolateral amygdala (BLA). Cholera Toxin B retro-labeling showed larger engagement of nucleus accumbens-projecting neurons in the BLA with 10-Hz phasic activation, and larger engagement of central amygdala projecting cells with 25-Hz tonic light. These outcomes argue that the LC activation patterns differentially influence both target networks and behavior.

Locus coeruleus (LC) adrenergic neurons are proposed to fire in 2 modes, phasic and tonic, with important functional consequences (Aston-Jones and Cohen 2005). However, how phasic and tonic LC activation differentially modulate learning and sensory valence encoding are not well understood. The advent of optogenetic techniques would appear to make it straightforward to test this idea, however, the literature on natural LC firing suggests the phasic/tonic distinction may be somewhat simplistic and, given LC output data, not so readily testable with optogenetic stimuli. Despite the often poor-matching of optogenetic patterns and LC firing, described below, differences in optical activation frequencies and patterns produce distinct behavioral outcomes. Here we characterize differing outcomes of phasic and tonic LC activations in acquisition of olfactory learning, spontaneous behavior and conditioned olfactory responses. Differing optogenetic patterns and/or frequencies, at the same site, redirected LC output to support the behavioral differences we observed. We suggest how LC optogenetic input redirects LC output is a critical future question for illuminating LC function.

In the early 1980s, natural LC firing patterns were recorded in awake rats and monkeys (Foote et al. 1980). Although LC neurons in anesthetized animals are activated by noxious input, LC neurons in awake animals are sensitive to a wide range of sensory stimuli. Brain state gates these LC responses. In rats, mean LC firing in Hz was 0.02 (REM sleep); 0.69 (slow wave sleep); and 2.12 (awake). Under stressful conditions, LC neurons display tonic discharge rates of 3–6 Hz (Abercrombie and Jacobs 1987; Lechner et al. 1997). Firing rates in monkeys were similar. Sustained rates of 7–15 Hz were seen when monkeys watched a syringe of preferred juice. Sensory stimuli at 5-s intervals were associated with LC bursts up to 10 Hz with post-burst pause, or no LC firing, depending on arousal (Aston-Jones and Bloom 1981). Habituation was not observed, but see Herve-Minvielle and Sara (1995). Reduced LC excitability accompanied consummatory behavior. In the context of associative learning (Sara and Segal 1991) and odd-ball vigilance (Aston-Jones et al. 1994), LC responses depended on contingencies and behavior. Bursts increased to cues predictive of reward and disappeared with overtraining. Overall LC increases predicted correct decision-making in oddball tasks.

Although LC firing patterns are described as varying between tonic and phasic firing modes (Rajkowski et al. 1994), natural LC firing appears to be aperiodic with nuanced variations in rate as seen in raster plots (Aston-Jones and Bloom 1981; Herve-Minvielle and Sara 1995; Takeuchi et al. 2016), where spike groupings in doublet and triplet patterns are common. With optogenetic tools, the functional effects of differing frequency and patterns of LC activation have been investigated. Although periodic optogenetic activation is an imperfect tool for mimicking natural LC patterns, recent investigations with this approach have provided us with rich information on LC functionality.

In awake mice, Takeuchi et al. (2016) used optogenetics to evaluate LC’s role in novelty facilitation of memory consolidation. Rodents exposed to novel environments after training exhibit strengthened training memory, which was mimicked by LC activation. The LC neurons in both the familiar and novel environments exhibited aperiodic firing. The average response rate increased in the novel environment, from 0–3 to 0.6–6 Hz for the same cells. In the novel environment, 25% of LC spikes met burst criteria. Within burst firing rate averaged ~ 18 Hz with overall firing at 2 Hz. For optogenetic purposes, cells were considered light-activated if 1/3 light pulses elicited a spike. Phasic light pulses at 25 Hz for 1 s every 5 s led to all LC spikes occurring in bursts and an overall firing increase to 4 Hz in the example provided. Consolidation of the training memory was facilitated with such an optical LC stimulation pattern.

In anesthetized rats, Vazey et al. showed that tonic activation increased EEG arousal at a threshold of 10 Hz. Tonic at 3 Hz and phasic pulses averaging 1.5 Hz (12 Hz, 3 pulses every 2 s) did not produce arousal. This permitted elucidation of differing effects of non-arousing tonic and phasic activation in the modulation of somatosensory input. Phasic input increased cortical event-related potential, whereas tonic did not. Both phasic and tonic input enabled subthreshold responses to become suprathreshold, recruiting more somatosensory neurons in response to a sensory stimulus. Phasic also increased late activity in the subthreshold somatosensory neurons, mimicking those of salient painful inputs. Between light pulses, LC neurons continued with irregular firing, with overall firing unchanged. This contrasts with likely supra-arousal phasic activation in the awake rodent (Takeuchi et al. 2016) when it appeared all spikes occurred in bursts and overall firing increased. It also contrasts with Carter et al. (2010) who observed clear arousal promoting effects of 3-Hz LC activation in mice. Other studies suggest less alignment of LC spikes with optogenetic pulses in vivo. During 5-Hz tonic LC light activation in mice, average LC firing varies from 0 to 13 Hz with all cells averaging < 5 Hz (McCall et al. 2015). Ten-hertz tonic light activating central amygdala (CeA) input to the LC generated the same variable LC firing, with a portion of cells showing decreased firing. However, both 5 and 10-Hz tonic light in the LC promoted anxiety and aversion. In another report, using 20-Hz pulses for 7 s, LC spiking varied from 2 to 5 Hz (Bari et al. 2020). Here, the behavioral output was improved attention and response inhibition depending on target structures.

The fascinating aspect of optogenetic experiments with phasic and tonic patterns is that they can consistently bias behavior in such different ways. Without concomitant in vivo awake recording during light activation in a behavioral task, a highly challenging undertaking, it is not possible to assume a tight relationship between optogenetic pulses and LC neuronal spiking. It is also not surprising that periodic stimuli interact in labile ways with state-dependent aperiodic LC firing. Nonetheless, different light pulse frequencies and patterns elicit distinct behavioral outcomes, even with small subject samples. Characterizing the differing outcomes and uncovering the mechanistic links between optogenetic inputs and their behavioral sequelae will deepen our understanding of how LC functions. Here we demonstrate that LC output redirection is one such mechanism, leading to the proposal that optogenetic patterns may differentially recruit LC downstream ensembles.

## Materials and Methods

### Animals and Ethics Statement

Tyrosine hydroxylase (TH)-CRE homozygous male breeders (Sage laboratories) were bred with Sprague–Dawley female breeder rats (Charles River) for TH-CRE heterozygous offspring that were used in this study. Rats of both sexes were housed in a 12-h light/dark cycle and had ad libitum access to food and water unless during food deprivation for experiments. During food deprivation, each rat was given 20 g of regular rat chow/day and was monitored for body-weight and health status on a weekly basis. All experimental protocols followed the guidelines of Canadian Council of Animal Care and were approved by Memorial University animal care committee.

**Figure 1:**
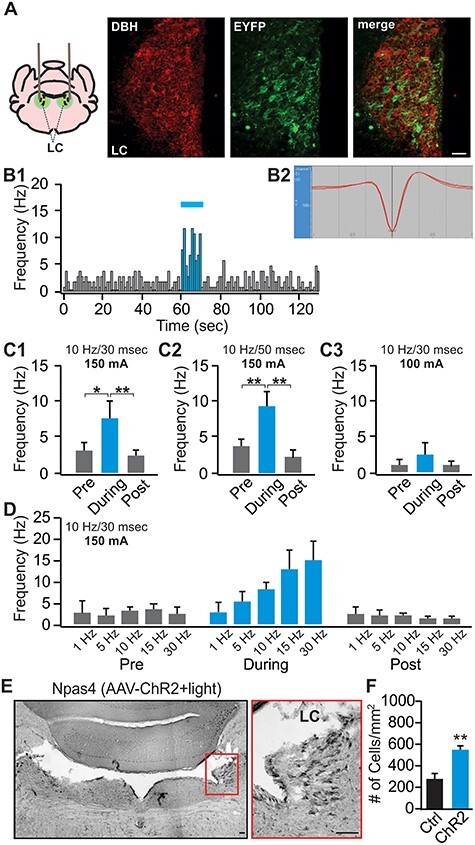
Validation of the light activation of the LC neurons. (*A*) Co-localization of DBH (red) and EYFP (green) in the LC of a TH-CRE rat infused with an AAV8-Ef1a-DIO-eChR2 (H134R)-EYFP. (*B*1) An example of an in vivo recording from the LC, showing increased firing of a LC neuron to a 10-Hz, 10-s light (30-ms duration and 150-mA intensity). (*B*2) The waveform of the recorded cell. (*C*1–*C*3) LC firing frequency changes induced by 10-s, 10-Hz light at 30-ms duration, 150 mA (*n* = 6, *C*1), at 50-ms duration, 150 mA (*n* = 6, *C*2), or at 30-ms duration, 100 mA (*n* = 4, *C*3). (*D*) LC responses to light activation with a range of frequencies at 150-mA intensity (*n* [1/5/10/15/30 Hz] = 2/3/12/6/6). (*E*) An example of Npas4 staining of the LC following light stimulation. Right panel is the zoom in of the red square on the left panel. Scale bars, 50 μm. (*F*) Npas4^+^ cell counts in the control (*n* = 6) versus ChR2 rats (*n* = 7). ^*^P < 0.05; ^*^^*^P < 0.01.

### Experimental Design and Statistical Analysis

**Figure 2:**
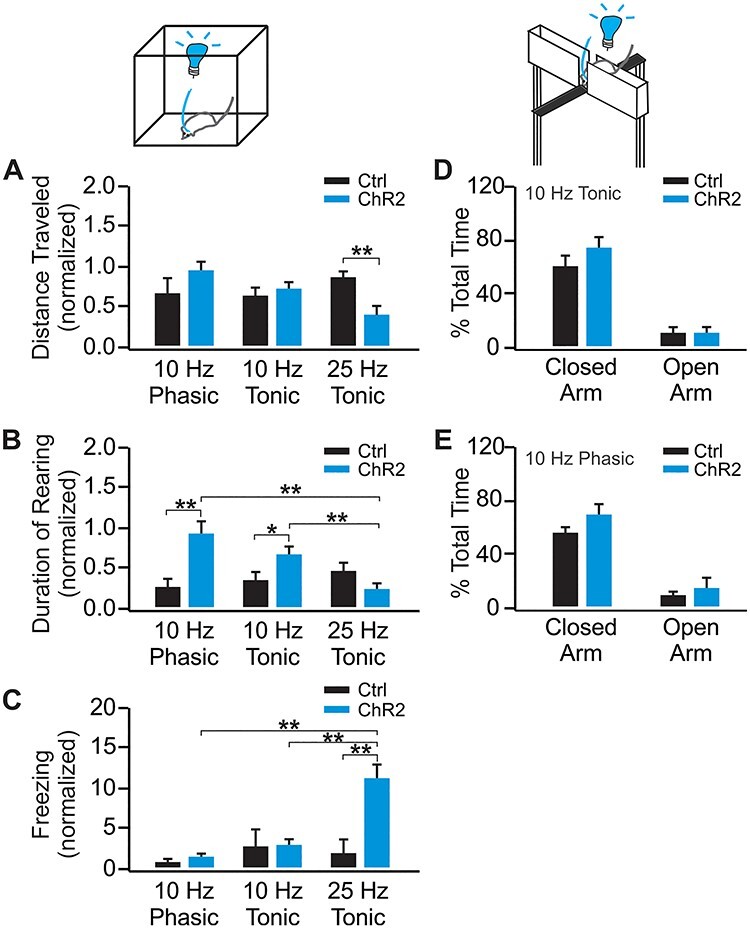
Ten-hertz phasic LC activation promotes exploration while 25-Hz tonic activation results in increased freezing and decreased mobility. (*A*) Distance traveled in the open field with various light patterns in the ChR2 (*n* = 7) and control (*n* = 7) rats, normalized to the baseline before the light stimulation. (*B*) Duration of rearing with various light patterns in the ChR2 (*n* = 7) and control (*n* = 7) rats. (*C*) Amount of freezing with various light patterns in the ChR2 (*n* = 6) and control rats (*n* = 7). (*D*) Percentage time spent in open and close arms of the EPM with 10-Hz tonic light activation in the ChR2 (*n* = 7) and control (*n* = 9) rats. (*E*) Percentage time spent in open and close arms of the EPM with 10-Hz long phasic light activation in the ChR2 (*n* = 6) and control (*n* = 6) rats. ^*^P < 0.05; ^*^^*^P < 0.01.

**Figure 3:**
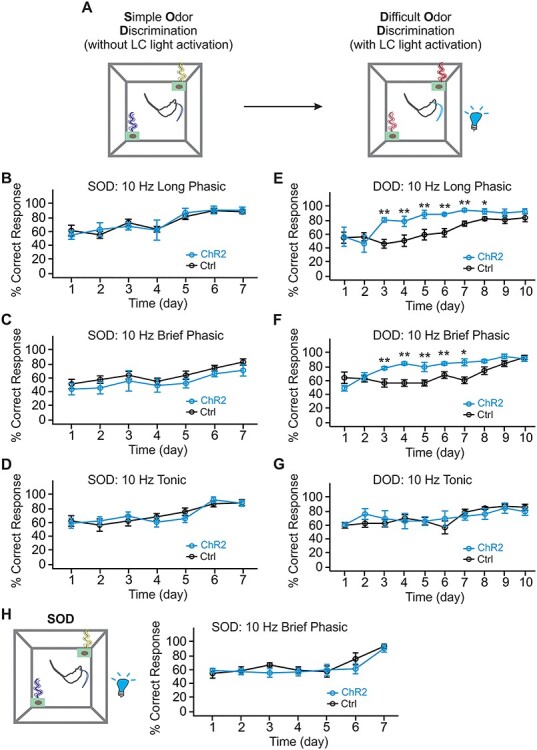
LC phasic patterns, but not tonic pattern, enhance similar odor discrimination learning, (*A*) Schematic of odor discrimination learning in rats. SOD learning without light is followed by DOD learning in the presence of various light patterns. (*B*) SOD training in rats of the 10-Hz long phasic groups (*n* [ChR2/Control] = 5/7). (*C*) SOD training in rats of the 10-Hz brief phasic groups (*n* [ChR2/Control] = 6/8). (*D*) SOD training in rats of the 10-Hz tonic light groups (*n* [ChR2/Control] = 6/7). (*E*) DOD training with 10-Hz long phasic light. (*F*) DOD training with 10-Hz brief phasic light. (*G*) DOD with 10-Hz tonic light. (*H*) SOD training with 10-Hz brief phasic light (*n* [ChR2/Control] = 6/7). ^*^P < 0.05; ^*^^*^P < 0.01.

**Figure 4:**
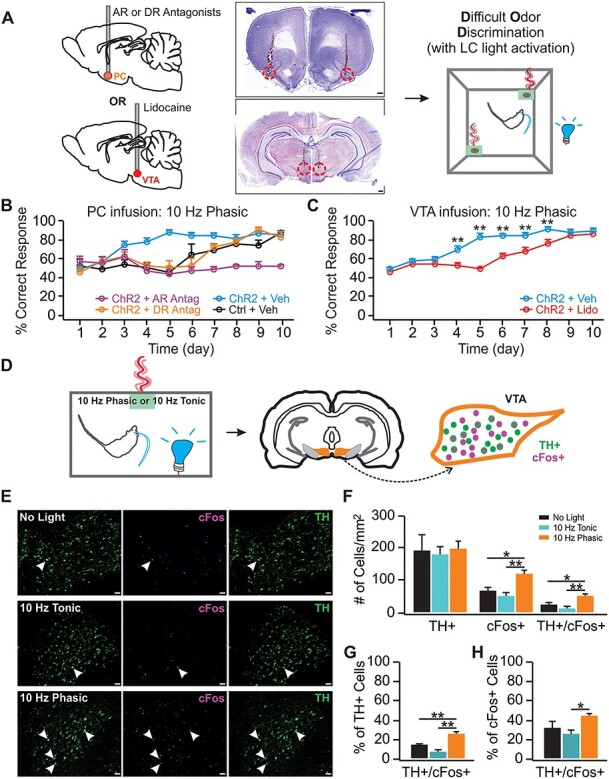
LC phasic activation engages VTA dopamine release to facilitate DOD, (*A*) Schematic of DOD training with cannular infusion with example targeting images. Scale bars: 500 μm. (*B*) DOD training with vehicle or drug infusions in the PC (*n* [ChR2 vehicle/Control vehicle/ChR2 AR block/ChR2 DR block] = 9/6/6/6). (*C*) DOD training with vehicle or lidocaine infusions in the VTA (*n* [lidocaine/vehicle] = 6/6). (*D*) Schematic of measuring cFos expression in the VTA with no-light control and different LC light patterns. (*E*) Examples images of cFos and TH staining in the VTA with no-light control (upper panel), 10-Hz tonic (middle panels) and 10-Hz phasic light (lower panels). Arrows indicated example TH^+^/cFos^+^ cells. Scale bars, 50 μm. (*F*) Total cFos^+^, TH^+^ and TH^+^/cFos^+^ cells activated in different groups (*n* [control/tonic/phasic] = 5/5/5). (*G*) Percentage TH^+^/cFos^+^ cells over total TH^+^ population. (*H*) Percentage TH^+^/cFos^+^ cells over total cFos^+^ population. AR: adrenoceptor; DR: dopaminergic receptor; Antag: antagonist.^*^P < 0.05; ^*^^*^P < 0.01.

**Figure 5:**
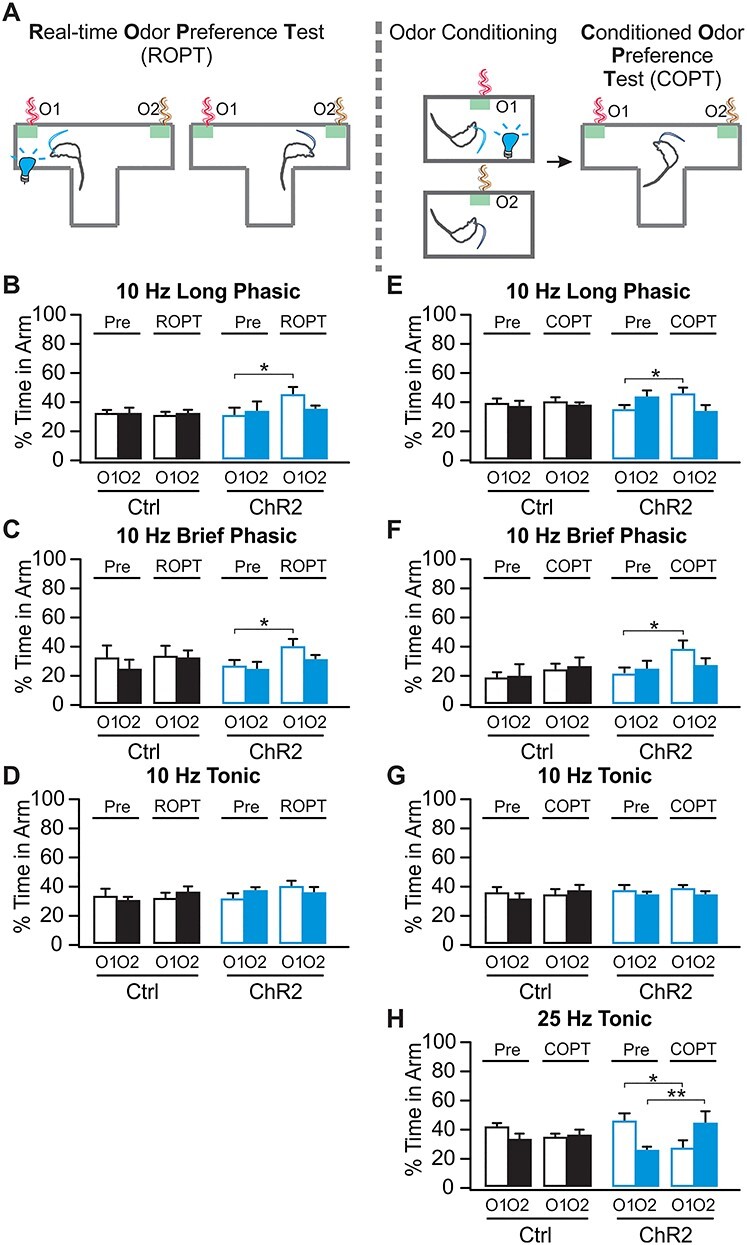
Twenty five-hertz tonic LC activation leads to conditioned odor aversion while 10-Hz phasic pattern results in odor preference, (*A*) Schematic of ROPT and COPT. (*B*) Percentage time spent in each odors in ROPT, with 10-Hz long phasic light paired with O1 (*n* [ChR2/Control] = 9/8). (*C*) Percentage time spent in each odors in ROPT with 10-Hz brief phasic light (*n* [ChR2/Control] = 6/8). (*D*) Percentage time spent in each odor in ROPT with 10-Hz tonic light (*n* [ChR2/Control] = 10/7). (*E*) Percentage time spent in each odor in COPT, with 10-Hz long phasic light conditioned with O1 (*n* [ChR2/Control] = 11/12). (*F*) Percentage time spent in each odor in COPT, with 10-Hz brief phasic light (*n* [ChR2/Control] = 6/7). (*G*) Percentage time spent in each odor in COPT with 10-Hz tonic light (*n* [ChR2/Control] = 11/10). (*H*) COPT with 25-Hz tonic light (*n* [ChR2/Control] = 8/11). ^*^P < 0.05; ^*^^*^P < 0.01.

**Figure 6:**
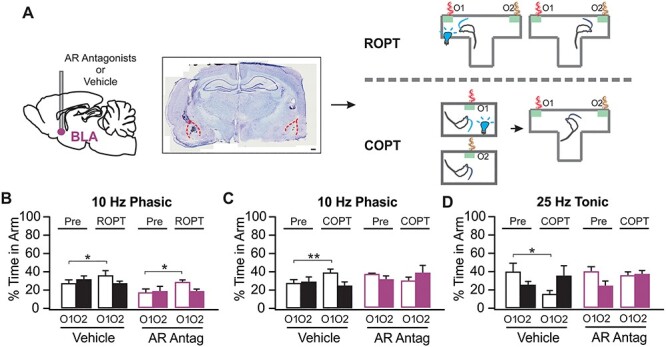
BLA ARs mediate the conditioned preference and aversion in COPT, (*A*) Schematic of brain infusion, followed by ROPT and COPT. An example targeting image of BLA is shown in the middle. Scale bar, 500 μm. (*B*) Percentage time spent in each odors in ROPT, with 10-Hz phasic light paired with O1 (*n* [Vehicle/AR antagonists] = 7/7). (*C*) Percentage time spent in each odor in COPT, with 10-Hz phasic light conditioned with O1 (*n* [Vehicle/AR antagonists] = 8/6). (*D*) COPT with 25-Hz tonic light (*n* [Vehicle/AR antagonists] = 8/6). AR: adrenoceptor. Antag: antagonist. ^*^P < 0.05; ^*^^*^P < 0.01.

**Figure 7:**
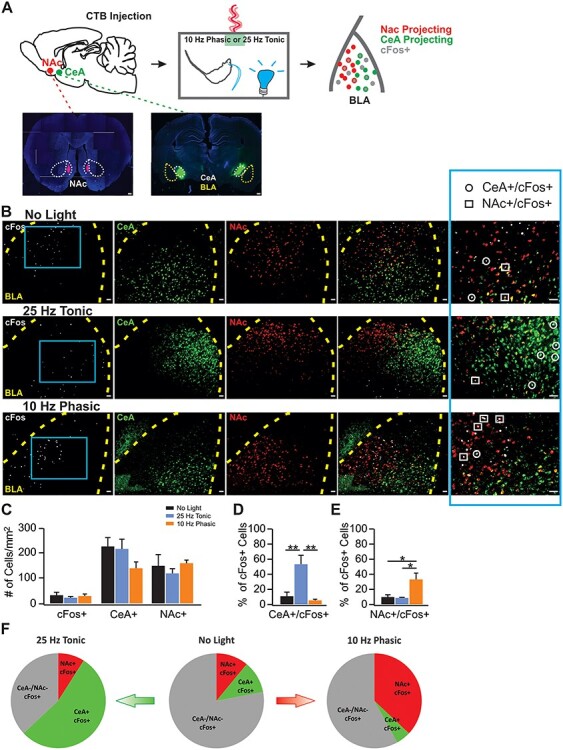
Ten-hertz phasic and 25-Hz tonic LC activation engage positive and negative projecting circuitry respectively in the BLA, (*A*) Schematic of measuring cFos activation in the BLA with CTB labeling NAc and CeA projecting neurons. (*B*) Examples images of cFos, CTB-488 (labeling CeA projecting neurons) and CTB-594 (labeling NAc projecting neurons) in the BLA in no-light control (upper panels), activated by 25-Hz tonic (middle panels) and 10-Hz phasic light (lower panels). Last column shows enlargement from the blue squares in the cFos images of the first column. Scale bars, 50 μm. (*C*) Total cFos^+^, CeA^+^ and NAc^+^ cells activated by tonic and phasic lights (*n* [control/tonic/phasic] = 4/3/3). (*D*) Percentage CeA^+^/cFos^+^ cells over total cFos^+^ population. (*E*) Percentage NAc^+^/cFos^+^ cells over total cFos^+^ population. ^*^P < 0.05. (*F*) Distributions of cFos^+^ cells in the BLA in no light (middle), 25-Hz tonic light (left) and 10-Hz phasic light (right) conditions.

Our study includes 4 major components, 1) establishment of in vivo LC optical activation using in vivo optrode recording and the Npas4 activity marker (Fig. 1); 2) the effects of phasic and tonic LC activations on general exploratory behavior and performance in the elevated plus maze (EPM; Fig. 2); 3) the effects of different LC activation patterns on difficult odor discrimination learning (Figs 3 and 4); and 4) the effects of different LC activation patterns on odor valence encoding (Figs 5–7). A total of 4 different light stimulation patterns were used in the behavioral tests, namely: 10-Hz long phasic (10 s every 30 s); 10-Hz brief phasic (300 ms every 2 s); 10-Hz tonic; and 25-Hz tonic. For general behavioral effects, odor discrimination, and valence learning, all patterns were explored (Figs 2, 3, and 5), except 25-Hz tonic was precluded in odor discrimination learning due to increased freezing and reduced mobility induced by this pattern. Similar effects of 2 LC phasic activity patterns were observed in all behavioral experiments. Therefore, subsequent mechanistic studies used one of the 2 patterns. For studying the role of ventral tegmental area (VTA) and piriform cortex (PC) dopamine (DA) in odor discrimination learning (Fig. 4), 10-Hz brief phasic and 10-Hz tonic patterns were used. For the effect of basolateral amygdala (BLA) adrenergic receptor (AR) blockade in odor valence learning (Fig. 6), 10-Hz long phasic and 25-Hz tonic light was used. For cFos activation in BLA (Fig. 7), the 10-Hz brief phasic effect was compared with 25-Hz tonic light.

One-way repeated analyses of variance (ANOVAs) were used in Figure 1*C* to compare the frequency changes pre-, during-, and post-light stimulation, followed by post-hoc Tukey tests. The 2 group comparison in Figure 1*F* was subjected to Student’s *t*-tests (unpaired, 2-tailed). Two-way mixed ANOVAs were used to compare the effects of different patterns of light on general behavior in Figure 2*A*–*C*, followed by post-hoc Tukey tests. *T*-tests (unpaired, 2-tailed) were used in Figure 2*D*–*E* for closed arm and open arm times separately. Two-way mixed ANOVAs followed by linear trend analyses were used to determine statistical significance for the odor discrimination experiments, followed by post-hoc tests between the control and ChR2 groups in Figures 3 and 4*B* and *C*. One-way ANOVA followed by post-hoc Tukey tests were used in Figure 4*F*–*H*. Two-way repeated ANOVAs were used to compare the 2 odor valences in the real-time odor conditioned test (ROPT) and conditioned odor preference test (COPT) in Figures 5 and 6, followed by post-hoc Tukey tests. One-way ANOVA followed by post-hoc Tukey tests were used in Figure 7. Data are presented as Mean ± standard error of the mean (SEM) in the graphs.

### Viral Transduction

An adeno-associated virus (AAVdj or AAV8) served as a vector to carry the genetic construct of channelrhodopsin 2 (ChR2) with a reporter gene for fluorescent proteins (EYFP or mCherry) under a double-floxed inverted open reading frame (DIO). Experimental constructs were AAVdj-EF1a-DIO-hChR2 (H134R)-mCherry or AAV8-Ef1a-DIO-eChR2 (H134R)-EYFP. The control construct was AAVdj-EF1a-DIO-mCherry. The Deisseroth Laboratory at Stanford University provided all AAVs.

### Stereotaxic Surgery

Three to 10-month-old adult TH-CRE rats received bilateral virus infusions (5E^+12^ vg/mL) in the LC under isofluorane anesthesia in a stereotaxic frame. Each hemisphere received 2 infusions, each of 0.7 μL (fluorescent beads: virus = 2:5) at the rate of 0.5 μL/min. The cannula was lowered at a 20° angle to avoid the transverse sinus. Infusion coordinates were 11.8–12.2-mm posterior, 1.2 and 1.4-mm bilateral, and 6.3-mm ventral with respect to bregma. At a minimum 1 month after infusion surgery, rats underwent optical fiber cannula (ferrule attached, containing optical fiber; Doric Lenses) implantation surgeries (11.8–12.2-mm posterior, 1.3-mm bilateral, and 6.3-mm ventral with respect to bregma), followed by a minimum of 2 weeks of recovery before commencing behavioral tests.

For experiments requiring drug infusion, metal infusion guide cannulas (23 gauge) were implanted in the BLA (AP: 2.5-mm posterior, ML: 4.9-mm bilateral, and DV: 7.8 mm; Carew et al. 2018), or PC (AP: 1.8–2.0-mm anterior, ML: 4-mm bilateral, and DV: 7.3–7.4 mm; Shakhawat et al. 2015), or VTA (AP: 5.3-mm posterior, ML: 1-mm bilateral, and DV: 8.1 mm; Rodriguez-Manzo and Canseco-Alba 2017; Nunes et al. 2019) combined with LC optical fiber cannula implantation. Internal cannula of 33 gauge (HRS Scientific) was used for infusion.

For experiments requiring Cholera Toxin B (CTB) infusions, surgeries were combined with LC optical fiber cannula implantation and rats were allowed a 10-day recovery before carrying out experiments. CTB-594 and CTB-488 (1% w/v in phosphate buffer; Invitrogen) were infused by separate 32g beveled 1-μL Hamilton syringes (Neuros 7001 KH) attached to a vertical infusion pump (Pump 11 Elite; Harvard Apparatus; Dong et al. 2017; McCall et al. 2017) in nucleus accumbens (NAc; 200 nL; AP: 1-mm anterior, ML: 1-mm bilateral, and DV: 6.5 mm) and CeA (150 nL; AP: 2.1-mm posterior, ML: 4.2-mm bilateral, and DV: 7.5 mm) respectively. Each infusion lasted 5 min, followed by a 5-min wait before withdrawing the syringe.

### In Vivo Electrophysiology

Rats underwent the in vivo electrophysiology experiments 1-month post-infusion. Rats were anesthetized with 15% urethane at 10 mL/kg of body weight and placed in a stereotaxic frame. The surgical procedure was carried out following appropriate sterilization. A hole was drilled in the skull (12.3-mm posterior and 1.3-mm left lateral to bregma) and an optrode, assembled just before the experiment (400-μm glass optical fiber; Thorlabs Inc), bundled with a 200/280-μm tungsten electrode; FHC), was lowered down at a 20° angle to 6.1–6.9-mm ventral to brain surface until LC neurons were identified by slow spontaneous spiking and burst response to toe pinch (audio-monitor and oscilloscope response; Quinlan et al. 2018). A glass optical fiber was connected to a laser diode fiber light source (Doric Lenses) by a mono-fiberoptic patch cord (0.48 NA, 400/430 μm). Blue light of 450 nm (90 mW; Doric Lenses) for a 400-μm core and 0.48-nA optical fiber was applied. Light pattern was controlled from Doric software. Following recording, rats were perfused with 4% paraformaldehyde (PFA). Brains were extracted and stored in 4% PFA overnight and then transferred to 25% sucrose solution (in 0.1M phosphate buffer) until sectioning for Nissl staining to locate the optrode placement. Only recordings that correctly targeted the LC were used for analysis.

Data analysis followed established procedures (Quinlan et al. 2018). Data were acquired and analyzed by SciWorks (DataWave/A-M Systems). Signal was detected at the lowest threshold of 1.5X amplitude of the background. Autosort protocol based on 6 dimensions (peak time, peak amplitude, valley 1 amplitude, valley 2 amplitude, and 2 principle components) was used to isolate similar cellular waveforms and cluster them in a cell-specific manner. Only clusters with LC-spike characteristics (e.g., broad action potentials; Quinlan et al. 2018) were further analyzed. Frequency histograms were generated to compare the firing rates of LC cells before, during, and after each light protocol.

## Behavioral Tests

### Light Stimulation for Behavioral Experiments

Two to 4 weeks after optical cannula implantation surgery, rats underwent behavioral tests. Bilateral photostimulation at 450 nm (20 mW/mm^2^ at fiber tip) was delivered by 2 laser light sources (LDFLS_450; Doric Lenses) through mono-fiberoptic patch cords. Current equivalence of power was 150 mA. Different patterns of stimulation were controlled from Doric software. Behavioral sessions were video-recorded by ANY-Maze software (Stoelting) and analyzed offline. Subsets of experiments were analyzed by persons who were blind to the experimental conditions.

### Drug Infusion

Drugs or vehicle were infused 30 min before behavioral testing through a 10-μL Hamilton syringe and infusion pump. Lidocaine (4%; Sigma) was infused in the VTA (0.3 μL/hemisphere over 3 min with an additional 1-min wait before withdrawing the syringe; Nunes et al. 2019). For PC and BLA infusions, 1 μL of drug or vehicle was infused per hemisphere over 2 min followed by a 1-min wait before withdrawing the syringe. The D1/5 receptor antagonist SCH 23390 (3.47 mM, Sigma), the α-AR antagonist phentolamine hydrochloride (10 mM; Sigma), and the β-AR antagonist alprenolol hydrochloride (120 mM; Tocris Bioscience; Shakhawat et al. 2015; Kempadoo et al. 2016) were used for PC and BLA infusions.

### General Behavioral Experiments

#### Open Field Maze

Rats explored an opaque plexiglass box (60 × 60 × 40.5 cm) with a black bottom for a 10-min daily session for 4 consecutive days while receiving no stimulation on day 1, 10-Hz long phasic stimulation on day 2, 10-Hz tonic stimulation on day 3, and 25-Hz tonic stimulation on day 4. Distance traveled, time spent rearing (both free and supported), and time spent freezing were recorded and analyzed. Freezing was counted as no body movement except breathing.

#### Elevated Plus Maze

Following a 15-min photo-stimulation in the home cage, rats were placed in the center of an EPM (50 × 10 cm each arm, 38-cm wall on the closed arms, 11 × 11-cm central platform, 52-cm high from the ground, inside painted black) facing the open arm opposite to the experimenter. Rats spent 5 min in the EPM while the stimulation continued. Time spent in closed and open arms was recorded.

### Olfactory Behavioral Experiments

#### Odorants

Odors used in the experiments are listed in Table 1. The odor concentrations for similar odor discrimination and valence tests were chosen either based on previous publication (Shakhawat et al. 2015) or estimated vapor pressure of 1 Pascal (Devore et al. 2013).

**Table 1:** The odorants used in various behavioral experiments

Name of the test	Odor 1 (O1; associated with photostimulation)	Odor 2 (O2)
Dissimilar odor discrimination	Almond extract	Coconut extract
Similar odor discrimination	Heptanol and Octanol (40:60 mixture; 0.001%)	Heptanol and octanol (50:50 mixture; 0.001%)
Valence test (10-Hz phasic)	Vanilla (2%)	Peppermint (2%)
Valence test (10-Hz tonic)	Orange (2%)	Propanoic acid (0.033%)
Valence test (25-Hz tonic)	Benzaldehyde (0.05%)	Isoamyl acetate (0.05%)

#### Odor Discrimination Learning

Rats were food deprived for 4–7 days before the onset of the experiments and food deprivation continued during the course of the experiment. Five days of habituation for context (box [60 × 60 × 40.5 cm], sponge and positive reinforcement [chocolate cereal]) were conducted first. Afterwards rats performed an odor discrimination task consisting of 10 trials/day, each trial being a maximum of 3 min. Two sponges containing odor 1 (O1) and odor 2 (O2), respectively were presented randomly in 2 corners of the box. The O1 sponge had a 2-cm^2^ hole at the top center, containing a retrievable chocolate cereal. To balance the smell of the chocolate cereal, a non-retrievable chocolate cereal was placed in a hidden hole in the O2 sponge. Between trials rats were confined to a “home corner” in the box with an L-shaped plexiglass barrier for 20 s while sponge positions were changed. During trials rats were allowed to explore the box and sniff the sponges. Trials ended as soon as a nose poke was made inside the hole, irrespective of the odor identity. The response was considered correct if nose poke was in O1 sponge. Trials in which no nose-poke occurred within 3 min were excluded from analysis. Percentage of correct responses was counted as the number of correct responses over the number of total nose pokes. Light stimulation was given only during trials and terminated at nose-poke, but not during intertrial intervals.

Dissimilar odor discrimination continued for a minimum of 7 days until rats reached 2 consecutive days of 80% success rate, followed by similar odor discrimination for 10 days.

#### Odor Valence Tests

For the COPT, the innate preference for 2 odors were first measured in a T-maze, then one of the odors was paired with LC light activation and preference for the 2 odors were tested again in the T-maze. Rats underwent a single 30-min session of habituation in a T-maze (long arm 183 × 19 cm, neutral arm 19 × 19 cm; and 20.5-cm high wall) on day 1. On day 2, O1 and O2 sponges were placed in 2 opposing arms, with positions counter-balanced between morning and afternoon sessions. Time spent in the arms corresponding with O1 and O2 during a 10-min session was recorded. On day 3, rats were confined to the O1 arm for 10 min in the morning with light stimulation; and to the O2 arm in the afternoon without light stimulation. The odors were switched in the arms on day 4 and the same conditioning as day 3 was repeated. Rats were trained for 1 trial or 3 trials (repeating procedures as in day 4–5 three times) and data were pooled. On the testing day, arm time was measured in morning and afternoon sessions with O1 and O2 sponges positioned in the exact same manner as day 2.

For the ROPT, rats were placed in a T-maze with 2 odorized arms and allowed to explore freely. Baseline preference for 2 odors was first recorded without light stimulation. During ROPT, LC light stimulation was associated with rats’ presence in one odorized arm, but not in the other. A 122-cm long arm was used. Day 1 habituation and day 2 baseline odor responses were conducted in the same manner as in COPT. On day 3, rats explored the maze freely for two 10-min sessions in the morning and afternoon. Light stimulation started upon a rat entering the O1 arm and stopped upon rat leaving the O1 arm. Odor positions were switched between morning and afternoon sessions.

#### CFos and Npas4 Induction Experiments

For cFos induction in Figures 4 and 6, rats were habituated to the experimental environment for 2 days. On the third and fourth days, rats were optically stimulated with either phasic or tonic patterns in their home cages, for 10 min/day, while being exposed to an odorized sponge (benzaldehyde 0.05%). Control rats were exposed to the odor only without light stimulation. Ninety min following the odor+light, or odor only stimulation on the fourth day, rats were anesthetized, perfused, and brains were collected.

For Npas4 induction to validate light activation in Figure 1, rats were habituated to the experimental environment for 1 day. Next day they were perfused 30-min following light stimulation in their home cage.

### Immunohistochemistry and Histology

Rats underwent trans-cardiac perfusion with cold isotonic saline followed by 4% PFA. Brains were extracted and kept in 4% PFA. Brains were then sectioned using a vibratome (Leica VT 1000P; Leica Biosystems) in 50-μm thick coronal slices and saved in a polyvinylpyrrolidone solution. For immunohistochemistry with Npas4 and DBH, slices were washed in PBS, and then incubated with primary antibodies.

Npas4 (1:500, Thermo Fisher Scientific) primary antibody mixed in phosphate buffer saline (PBS) with 2% normal goat serum and 0.2% Triton-X was applied for 3 nights at 4°C. Following a 3 × 10 min wash in PBS, a biotinylated anti-rabbit secondary antibody (1:1000; Vector laboratories, Burlingame, CA) was applied. After 2 h of secondary incubation and a 3 × 10 min wash, slices were incubated in an avidin-biotin complex for 1.5 h followed by a PBS wash. Slices were then placed in a solution containing 15-μL SG Gray chromogen (Vector laboratories) with 24-μL peroxide per ml of PBS. After optimum color development, slices were washed in PBS, dried overnight, dehydrated in graded ethanol, and mounted with permount.

For dopamine beta hydroxylase (DBH) staining, after a primary antibody (1:500, EMD Millipore) incubation, slices were incubated with a fluorescence conjugated anti-mouse secondary antibody (1:1000; Invitrogen/Thermo Fisher Scientific) at room temperature for 2 h, followed by cover-slipping with Vectashield antifade mounting medium (Vector laboratories).

For cFos and TH co-labeling, 50-μm free floating sections from phasic and tonic stimulated rats, belonging to similar positions in the anterior–posterior axis of the brain as determined by unaided visual observation, were chosen in an unbiased manner. The sections were washed in Tris buffer (0.1 M, pH 7.6) twice for 10 min each, followed by 10 min in Tris A (0.1% TritonX in Tris buffer), and Tris B (0.1% TritonX and 0.005% BSA in Tris buffer) before applying a blocking solution of 10% normal goat serum (Sigma-Aldrich) for 1 h. This was followed by a 10-min wash each in Tris A and Tris B before incubating in 1:2000 primary antibody solution prepared in Tris B at 4 **°**C (TH, EMD Millipore; cFos, Cell Signaling). After 2 nights, sections were washed for 10 min each in Tris A and Tris B and incubated in a 1:1000 secondary antibody solution prepared in Tris B at 4 **°**C (anti-rabbit Alexa 647, anti-mouse Alexa 488; Invitrogen/Thermo Fisher Scientific). This was followed by 10-min washes in Tris A, Tris D (0.1%Triton X and 0.005% BSA in 0.5 M Tris buffer), and Tris buffer, respectively. Finally, sections were mounted with antifade mounting medium.

Nissl staining was done by rehydrating the slides in graded ethanol, incubating in 0.5% cresyl violet for 8 min, washing in distilled water for 1 min, and then dehydrating in graded ethanol. After a 5-min xylene step, slides were coverslipped with permount.

### Image Acquisition and Analysis

Images were acquired by an EVOS 5000 (Thermo Fisher Scientific), and a BX-51 (Olympus) for fluorescent and bright-field images. Images were acquired similarly for phasic and tonic stimulated rats, keeping gain and exposure time the same throughout each experiment. Images were analyzed using ImageJ software. Images underwent background subtraction before manual cell counting. For LC activation success, the number of Npas4^+^ cells was counted. For BLA and VTA activation, cFos^+^ cells and double-labeled CTB cells were counted. Three to 6 images per animal were analyzed and values from both hemispheres were averaged. A subset of the images was analyzed blindly.

## Results

### Validation of Light Activation of LC Neurons

Three weeks following LC AAV infusion (for targeting see Supplementary Fig. S1), we observed LC ChR2 uptake marked by the fluorescence reporter EYFP (Fig. 1*A*) or mCherry. All rats examined expressed the reporter bilaterally in the LC. The percentage of LC neurons (DBH expressing cells) transfected with ChR2 (co-expressing EYFP or mCherry) were 73.7 ± 12.8% (*n* = 8), and 92.3 ± 2.7% ChR2 cells were DBH^+^. We conducted in vivo optrode LC recordings. Figure 1*B*1 and *B*2 shows an LC neuron activated by a 10-s, 10-Hz light train (30-ms pulses, laser intensity 150 mA). Figure 1*C*1–*C*3 shows LC activation by 10-Hz trains with 2 light intensities and pulse widths. The LC firing frequency increases were significantly induced by the 10-s, 10-Hz light at 30-ms duration, 150 mA (*F*_2,10_ = 8.69, *P* = 0.006, *n* = 6; Fig. 1*C*1), or 10 Hz at 50-ms duration, 150 mA (*F*_2,10_ = 25.42, *P* < 0.001, *n* = 6; Fig. 1*C*2), but not at 30-ms duration, 100 mA (*F*_2,6_ = 2.48, *P* = 0.16, *n* = 4; Fig. 1*C*3). The 30-ms, 150-mA light that effectively activated LC firing was used subsequently in recording (Fig. 1*D*) and behavioral experiments. Increasing light frequency elevated LC firing in ~linear fashion in the frequency range < 30 Hz (Fig. 1*D*), with a firing output up to 15 Hz. Although average LC firing rates with 5- and 10-Hz light approximate activation rates, we found LC neurons were not individually driven at those rates in vivo. Similar outcomes were observed in mouse in vivo where average LC firing with light activation was made up of LC neuron responses of widely varying frequencies (McCall et al. 2015). A caveat is that LC output from optrode recording is not an accurate estimate of the average effects throughout the LC, as the electrode tip is in close proximity to the tip of the optical fiber where light intensity is maximal. Therefore, average LC output during the light stimulation is likely lower than the output reported here. The immediate early gene Npas4 revealed light-induced expression in LC (*t* = 4.53, *P* < 0.001, *n* [ChR2/Control] = 6/7; Fig. 1*E* and *F*). Recordings with the optrode placements outside the LC did not respond to the light (an example see Supplementary Fig. S2).

### Different Patterns of LC Activation Show Distinct General Behavioral Effects

First we investigated whether phasic and tonic LC activations have differential effects on locomotion, exploratory behavior, and stress. We included 4 patterns including 2 phasic patterns: 10-Hz long phasic (10 s every 30 s), and 10-Hz brief phasic (300 ms every 2 s). These patterns are consistent with recent studies in terms of frequency range and duration (Carter et al. 2010; Kempadoo et al. 2016; Vazey et al. 2018). The 10-Hz brief phasic pattern mimics physiological LC firing in response to environmental stimuli (Aston-Jones and Bloom 1981; Nakamura et al. 1987). The 2 tonic patterns are 10-Hz tonic and 25-Hz tonic, corresponding to the LC output in the range of 10–15 Hz in our in vivo recording (Fig. 1*D*).

The effects of these LC activation patterns on open field distance traveled, duration of rearing, and freezing were tested (Fig. 2*A*–*C*). In the open field experiments, rats underwent a series of tests in a fixed order: baseline without light activation, 10-Hz long phasic, 10-Hz tonic, and 25-Hz tonic. Data generated by different light patterns were normalized to the same baseline parameters for comparisons. A subset of rats underwent baseline measurements followed by 10-Hz brief phasic light activation (see Supplementary Fig. S3). Ten-hertz phasic and tonic stimulated rats showed increased rearing while 25-Hz rats showed less mobility and increased freezing.

For distance traveled, the baselines in the ChR2 (24.70 ± 3.10 m; *n* = 7) and control groups (19.71 ± 4.30 m; *n* = 7) are similar (*t* = 0.916, *P* = 0.376). However, there is a significant Light Pattern X Group interaction during light stimulations (*F*_2, 24_ = 8.831, *P* = 0.001; Fig. 2*A*). The ChR2 group showed a reduction in distance traveled with 25-Hz light compared with the control group (*t* = 4.02, *P* = 0.002). Duration of rearing was significantly different among groups (*F*_2, 24_ = 13.617, *P* < 0.001, *n* [ChR2/Control] = 7/7; Fig. 2*B*). The ChR2 group showed increased rearing with the 10-Hz long phasic light (*t* = 6.449, *P* < 0.001) and the 10-Hz tonic light (*t* = 3.015, *P* = 0.043) compared with controls. Significant effects were also observed between the 10-Hz long phasic and 25-Hz tonic light (*t* = 7.908, *P* < 0.001), and between the 10-Hz tonic and 25-Hz tonic light (*t* = 4.847, *P* = 0.006) in the ChR2 groups. The amount of freezing also differed among groups (*F*_2, 22_ = 15.759, *P* < 0.001, *n* [ChR2/Control] = 6/7; Fig. 2*C*). The ChR2 group with 25-Hz tonic light showed increased freezing compared with the control group (*t* = 7.903, *P* < 0.001), 10-Hz long phasic (*t* = 8.002, *P* < 0.001), and 10-Hz tonic (*t* = 6.749, *P* = 0.001) light activation. A separate test of the 10-Hz brief phasic light with a corresponding light control group showed increased duration of rearing (*t* = 2.232, *P* = 0.038; see Supplementary Fig. S2), similar to that of the 10-Hz long phasic light.

Anxiety during tonic and phasic 10-Hz light stimulations was measured in an EPM (Fig. 2*D* and *E*), and no differences were observed in the light-activated and control groups. For 10-Hz tonic, there was no difference between the ChR2 and the control group in time spent in either the closed arms (*t* = 1.383; *P* = 0.188) or the open arms (*t* = 0.101, *P* = 0.921, *n* [ChR2/Control] = 7/9; Fig. 2*D*). Similarly, neither the time spent in the closed arms (*t* = 1.439; *P* = 0.181), nor in the open arms (*t* = 0.838, *P* = 0.421), was significantly different between ChR2 and control groups with 10-Hz long phasic light activation (*n* [ChR2/Control] = 6/6; Fig. 2E). Together, these results suggest only 25-Hz tonic light induced a stress phenotype while 10-Hz tonic LC activation did not result in anxiety or stress in either the open field or EPM.

### LC Phasic Patterns Enhance Similar Odor Discrimination Learning

Rats were trained to associate a food pellet with one odor from an odor pair (Fig. 3*A*). After learning the simple odor discrimination (SOD) with a dissimilar odor pair in the absence of light activation (Fig. 3*B*–*D*), rats then learned the DOD with a similar odor pair with LC activation (Fig. 3*E*–*G*). Three light patterns were used during DOD training: 10-Hz long phasic (Fig. 3*B* and *E*), 10-Hz brief phasic (Fig. 3*C* and *F*) and 10-Hz tonic (Fig. 3*D* and *G*). The 25-Hz tonic stimulation that induced significant freezing was not included in this learning paradigm. While both phasic patterns facilitated DOD learning, 10-Hz tonic light activation had no effect on DOD learning.

ChR2 and control rats showed similar learning in the initial SOD. All groups showed improved performance over time (*F*_6, 60_ = 10.99, *P* < 0.001 for 10-Hz long phasic, *n* [ChR2/Control] = 5/7; Fig. 3*B*; *F*_6, 72_ = 9.125, *P* < 0.001 for 10-Hz brief phasic, *n* [ChR2/Control] = 6/8; Fig. 3*C*; *F*_6, 66_ = 12.097, *P* < 0.001 for 10-Hz tonic light, *n* [ChR2/Control] = 6/7; Fig 3*D*), but there were no group differences in all light patterns**.** However, in DOD, both brief and long phasic LC activations accelerated learning acquisition. For long phasic 10-Hz LC activation, there was a significant Day X Group interaction (*F*_9, 90_ = 2.880, *P* = 0.005), and a Group effect (*F*_1, 10_ = 10.069, *P* = 0.010; Fig 3*E*). Better performance in the ChR2 group was observed on days 3–8 (*P* < 0.05 or *P* < 0.01). Similarly, for DOD training with 10-Hz brief phasic light, a significant Day X Group interaction (*F*_9, 108_ = 4.429, *P* < 0.001) and a Group effect (*F*_1, 12_ = 17.47, *P* = 0.001; Fig 3*E*) were observed. Better correct response rates were observed from days 3–7 with this 10-Hz brief phasic light pattern (*P* < 0.05 or *P* < 0.01). In contrast, tonic 10-Hz LC activation did not alter acquisition. There was no effect of Day X Group interaction (*F*_9, 99_ = 0.983, *P* = 0.459), or Group (*F*_1, 11_ = 0.033, *P* = 0.860). Linear trend analysis showed improvement with time in both ChR2 (*F*_1, 5_ = 12.810, *P* = 0.016) and control groups (*F*_1, 6_ = 61.491, *P* < 0.001; Fig 3*F*).

We then tested whether LC phasic activation also improves SOD. Our result showed that SOD learning was not affected by phasic 10-Hz LC activation (*n* [ChR2/Control] = 6/7; Fig 3*H*). There was no Group X Day interaction (*F*_6, 66_ = 0.755, *P* = 0.608), or Group effect (*F*_1,11_ = 0.527, *P* = 0.483). Latency to nose poke showed reduction with training, and was not different between the control and ChR2 groups (see Supplementary Fig. S4). This underscores norepinephrine (NE’s) role in difficult discriminations that require pattern separation (Shakhawat et al. 2014, 2015). Enhancement of subtle tactile discriminations by tonic 5-Hz LC activation (Rodenkirch et al. 2019) has also been reported in rats, a frequency not explored here.

### LC–VTA–PC DA Circuitry Mediates the Facilitating Effect in Similar Odor Discrimination Learning

Increased extracellular DA in the hippocampus following LC stimulation has been shown to be critical in spatial learning (Kempadoo et al. 2016) and novelty-mediated memory consolidation (Takeuchi et al. 2016). We next tested the potential involvement of NE and DA in the PC upon LC 10-Hz brief phasic light activation in DOD (Fig. 4*A*). The DOD was prevented when a mixture of α1-AR antagonist phentolamine and β-AR antagonist alprenolol were infused in the PC before training, however, the D1/5 receptor antagonist SCH 23390 infusion selectively abolished LC phasic light induced learning facilitation (*n* [ChR2 vehicle/Control vehicle/ChR2 AR block/ChR2 DR block] = 9/6/6/6); Fig. 4*B*), but not DOD acquisition. There was a significant Day effect (*F*_9, 207_ = 15.98, *P* < 0.001), a Group X Day interaction (*F*_27, 207_ = 5.196, *P* < 0.001), and a Group effect (*F*_3, 23_ = 17.73, *P* < 0.001). A significant difference in learning was observed between the D1/5 antagonist group and ChR2 vehicle group (*t* = 8.624, *P* < 0.001), and the AR antagonist group (*t* = 8.091, *P* < 0.001), and between the AR antagonist group and the ChR2 vehicle group (*t* = 17.488, *P* < 0.001). However, the D1/5 antagonist group performed similarly to the non-ChR2 control vehicle group (*t* = 1.421, *P* = 0.748).

These results argue that while NE in the PC is essential for pattern separation-dependent odor discrimination to occur, DA release in the PC during LC phasic light contributes a learning facilitating effect. There are 2 possible scenarios: either LC axon terminals co-release DA or increase extracellular DA through other mechanisms upon LC phasic activation (Kempadoo et al. 2016; Takeuchi et al. 2016), or VTA releases DA into the PC (Datiche and Cattarelli 1996; Aransay et al. 2015) upon LC activation. To determine the source of DA released during odor discrimination learning, we infused lidocaine into the VTA to silence the VTA during DOD. Lidocaine infusion prevented the learning facilitation effects of the LC phasic light (*n* [lidocaine/vehicle] = 6/6; Fig. 4*C*), but not the acquisition of DOD learning. There was a significant Day effect (*F*_9, 90_ = 41.46, *P* < 0.001), a Group X Day interaction (*F*_9, 90_ = 5.60, *P* < 0.001), and a Group effect (*F*_1, 10_ = 90.94, *P* < 0.001)**.** Higher correct response rates of the ChR2 vehicle group relative to the ChR2 lidocaine group on days 4–7 were observed (*P* < 0.01). However, the performance of the 2 groups on the last 3 days (8–10) was comparable (*P* > 0.05).

Why does phasic LC activation promote DOD while tonic activation at the same frequency does not? In other words, does LC phasic activation engage DA neurons in the VTA more effectively than tonic activation? We next studied neuronal activation patterns in the VTA induced by phasic versus tonic LC activations. We measured cFos expression in the VTA following odor exposure only (no-light control), 10-Hz brief phasic or 10-Hz tonic LC activation paired with an odor (*n* [control/tonic/phasic] = 5/5/5; Fig. 4*D*). The phasic light increased overall cFos activation in the VTA, as well as the portion of activated TH^+^ cells (Fig. 4*E*–*G*). Despite the similar numbers of TH^+^ cells in the 3 groups (*F*_2,12_ = 0.062, *P* = 0.940), there were different proportions of cFos^+^ cells (*F*_2,12_ = 11.633, *P* = 0.002) and TH^+^/cFos^+^ cells (*F*_2,12_ = 10.436, *P* = 0.002; Fig. 4*E* and *F*). The phasic pattern activated significantly more cFos^+^ cells in the VTA than the no-light control (*t* = 4.649, *P* = 0.017) and the tonic pattern (*t* = 6.647, *P* = 0.001), and generated a larger number of TH^+^/cFos^+^ cells compared with the control (*t* = 4.598, *P* = 0.017) and the tonic activation (*t* = 6.230, *P* = 0.002). The percentage of cFos^+^ cells in the total TH^+^ population is significantly higher with the phasic pattern than with the no-light control and the tonic pattern (*F*_2,12_ = 33.984, *P* < 0.001; Fig. 4*G*). The percentage of TH^+^/cFos^+^ cells over total cFos^+^ cells is also higher in the phasic group (*F*_2,12_ = 4.938, *P* = 0.027; Fig. 4*H*). These results argue that the 10-Hz LC phasic pattern engages VTA DA neurons whereas the 10-Hz tonic pattern does not. This may explain the learning facilitation effect of PC DA with phasic, but not tonic, LC activation.

### LC Phasic and Tonic Patterns Promote Differential Odor Valence Learning

Besides differentially modulating discrimination learning by different LC activation patterns, tonic LC activation is involved in stress and aversive learning (Hirschberg et al. 2017; McCall et al. 2017; Llorca-Torralba et al. 2019). However, the effect of phasic LC activation on adult valence encoding is unknown. We next tested the intriguing possibility that tonic and phasic LC activations could differentially mediate valence learning. Tonic LC optical stimulation has been associated with real-time place aversion and conditioned place aversion (McCall et al. 2015, 2017). Here, we employed similar tests with odors using ROPT and COPT (Fig 5*A*).

In the ROPT, rats were tested for the time spent in the 2 odorized zones, first in the absence, then in the presence, of light activation associated with one odor (O1). Ten-hertz long phasic activation increased the time ChR2 rats spent in the light-paired odor zone (*n* [ChR2/Control] = 9/8; Fig. 5*B*). A significant Odor X Time interaction was observed in the ChR2 group (*F*_1,8_ = 8.269, *P* = 0.021), but not in the control group (*F*_1,7_ = 0.080, *P* = 0.786). ChR2 rats spent significantly more time in O1 during the 10-Hz phasic light, compared with the baseline (*t* = 3.35, *P* = 0.045). With 10-Hz brief phasic activation, there was a significant effect of Time in the ChR2 rats (*F*_1,5_ = 12.490, *P* = 0.017, *n* [ChR2/Control] = 6/8; Fig. 5*C*). ChR2 rats spent more time in O1 during light stimulation compared with baseline (*t* = 4.341, *P* = 0.028). In contrast, 10-Hz tonic light had no significant effect in the ChR2 group (*F*_1,9_ = 2.587, *P* = 0.142, *n* [ChR2/Control] = 10/7; Fig. 5*D*).

In the COPT, rats were light stimulated in the presence of one odor, O1. Time spent with O1 versus a control odor O2, before and after conditioning was compared. There was a conditioned preference with both 10-Hz phasic patterns, but not 10-Hz tonic pairing. 10-Hz long phasic activation resulted in a preference for O1 in the ChR2 group (*F*_1,10_ = 6.622, *P* = 0.028), but not in the control group (*F*_1,11_ = 0.034, *P* = 0.857, *n* [ChR2/Control] = 11/12; Fig. 5*E*). ChR2 rats spent significantly more time in O1 (*t* = 3.44, *P* = 0.035) after odor conditioning. Similarly, 10-Hz brief phasic activation also induced preference for the light-conditioned O1 in the ChR2 group (*F*_1,5_ = 6.610, *P* = 0.049), but not the control group (*F*_1,6_ = 0.001, *P* = 0.975, *n* [ChR2/Control] = 6/7; Fig. 5*F*). ChR2 rats spent more time in O1 following odor conditioning (*t* = 3.889, *P* = 0.040). However, the 10-Hz tonic light had no effect on odor preference (*F*_1,10_ = 0.017, *P* = 0.899, *n* [ChR2/Control] = 11/10; Fig. 5*G*).

The immobility associated with the tonic 25-Hz light (Fig. 2*C*) precluded ROPT testing. In COPT, a conditioned avoidance was induced by the 25-Hz light-paired odor (*F*_1,7_ = 14.811, *P* = 0.006, *n* [ChR2/Control] = 8/11; Fig. 5*H*). ChR2 rats spent significantly less time in the O1 arm after it was associated with the 25-Hz tonic light, compared with the baseline (*t* = 4.23, *P* = 0.020), while they spent more time in the control odor O2 (*t* = 5.169, *P* = 0.008). This replicates the conditioned place aversion seen with tonic 5-Hz activation in mice (McCall et al. 2015) and with chemogenetic tonic LC activation in rats (Hirschberg et al. 2017).

### LC Phasic and Tonic Patterns Engage Differential BLA Circuitry in Odor Valence Learning

In mice, it has been shown that LC tonic activity promotes aversive behavior through BLA β-adrenoceptors (McCall et al. 2017). The BLA is a critical site for valence associative learning (O'Neill et al. 2018). The BLA has been found to contain functionally distinct neuronal populations projecting to either negative- (CeA) or positive- (NAc) valence encoding circuitry (Kim et al. 2016; Namburi et al. 2016). It is plausible that the LC–BLA projection is involved in the odor valence encoding observed here with differential LC light activation patterns.

Here we first tested the involvement of BLA ARs in ROPT and COPT with either 10-Hz long phasic or 25-Hz tonic LC activation (Fig. 6*A*). Increased time in the light stimulated odor zone with 10-Hz phasic light in ROPT was not affected by AR blockade (*n* [Vehicle/AR antagonists] = 7/7; Fig. 6*B*). Significant Time X Odor effects were observed in both vehicle-infused rats (*F*_1,6_ = 6.947, *P* = 0.039) and AR antagonist infused rats (*F*_1,6_ = 9.693, *P* = 0.021). Both vehicle (*t* = 3.730, *P* = 0.039) and AR antagonist groups (*t* = 4.088, *P* = 0.027) showed more time spent in the light-activated odor zone O1 during ROPT.

However, BLA α- and β-AR blockade with phentolamine and alprenolol prevented both LC 10-Hz long phasic light induced odor preference (*n* [Vehicle/AR antagonists] = 8/6; Fig. 6*C*) and LC 25-Hz tonic light induced odor aversion in COPT (*n* [Vehicle/AR antagonists] = 8/6; Fig. 6*D*). While the vehicle group developed odor preference with 10-Hz long phasic light (*F*_1,7_ = 7.220, *P* = 0.031) and spent significantly more time in the conditioned odor O1 during the COPT (*t* = 5.048; *P* = 0.009), the AR antagonist infused group did not (*F*_1,5_ = 2.778, *P* = 0.156; Fig. 6*C*). Similarly, the AR antagonist infused group showed no development of aversion with 25-Hz tonic LC activation (*F*_1,4_ = 3.297, *P* = 0.129), while the vehicle group showed a significant effect of time (*F*_1,7_ = 5.875, *P* = 0.046), and spent significantly less time in the conditioned odor O1 during the COPT (*t* = 4.309, *P* = 0.019; Fig. 6*D*).

Taken together, while BLA NE mediates conditioned valence learning dependent on differential patterns of LC activation, this circuitry is not involved in real-time preference in ROPT. Phasic light mediated enhanced exploration (Fig. 2*B*) may explain the acute ROPT effect of 10-Hz phasic light. This is consistent with a previous report that tonic LC mediation of real-time place aversion is not associated with the LC–BLA projections (McCall et al. 2017).

After establishing the requirement of BLA NE in LC light mediated valence learning in COPT, we next tested whether tonic and phasic activation of the LC biases activation of the BLA ensembles projecting to BLA–CeA aversive and BLA–NAc reward circuitry respectively. We infused retro-tracing dyes linked to CTB in the CeA and NAc, and examined the overlap of CeA or NAc projecting neurons with cFos^+^ cells in the BLA activated by odor only (no-light control), 10-Hz brief phasic or 25-Hz tonic LC lights (Fig. 7*A* and *B*). The CTB labeled CeA (*F*_2,7_ = 2.028, *P* = 0.202) and NAc (*F*_2,7_ = 0.340, *P* = 0.723) projecting cell numbers were comparable in the 3 groups (*n* (control/tonic/phasic) = 4/3/3; Fig. 7*C*). Intriguingly, although the 2 LC light patterns activated similar numbers of cFos^+^ cells in the BLA compared with the control (*F*_2,7_ = 0.888, *P* = 0.453; Fig. 7*C*), the distribution patterns of cFos^+^ cells were dramatically different (see example images in Fig 7*B*). The proportion of CeA^+^ cells that were cFos^+^ cells was significantly higher in the 25-Hz tonic group (*F*_2,7_ = 14.232, *P* = 0.003; Fig. 7*D*) compared with either the non-light control (*t* = 4.539, *P* = 0.008) or the 10-Hz brief phasic light (*t* = 4.796, *P* = 0.006). On the other hand, the proportion of NAc^+^ cells in cFos^+^ cells was significantly higher in the 10-Hz phasic light group (*F*_2,7_ = 10.648, *P* = 0.008; Fig. 7*E*) compared with either non-light controls (*t* = 4.033, *P* = 0.015) or the 25-Hz tonic light (*t* = 4.052, *P* = 0.015). The differential distributions of cFos^+^ cells in different groups are displayed in the pie charts (Fig. 7*F*). In no-light controls, equal amount of cFos^+^ cells (11%) were NAc and CeA projecting cells, whereas 37% were NAc projecting and 5% were CeA projecting with the 10-Hz brief phasic light, and 54% were CeA projecting and 9% were NAc projecting with the 10-Hz tonic light. A small portion of projecting cells (4.4%) expressed both CTBs, however, the activation of the double-CTB-labeled cells was very low (0.72%). Additionally, phasic and tonic light activations in the absence of an odor did not lead to a difference in cFos activation patterns (see Supplementary Fig. S5), consistent with a modulatory role of NE. Phasic and tonic modes of LC activations re-distributed neuronal ensembles activated by an odorant in the BLA. Selective activation of one valence encoding ensemble may inhibit the ensemble of the opposite valence in the BLA (Namburi et al. 2015). Taken together, the 10-Hz LC phasic pattern preferentially activates NAc projecting neurons in the BLA, whereas the 25-Hz tonic LC activation preferentially engages CeA projecting neurons.

VTA projections to the NAc facilitate BLA–NAc circuitry in promoting a reward-seeking response to sensory cues (Yun et al. 2004; Ambroggi et al. 2008). The 10-Hz phasic, but not 10-Hz tonic, LC activation, efficiently recruited VTA dopaminergic neurons in our study (Fig. 4*D*–*H*) suggesting that 10-Hz phasic LC activation likely engages the VTA–NAc pathway more efficiently compared with a tonic pattern. This is indeed the case when we compared the cfos activation in the VTA by 10-Hz phasic light versus 25-Hz tonic light (see Supplementary Fig. S6). More cFos^+^ cells were observed with 10-Hz phasic LC activation and a bigger portion of NAc projecting neurons were involved.

## Discussion

This work aimed to further our understanding of whether and how the LC produces activation mode-specific behavioral responses. We assessed how LC activation patterns differentially modulate general behavior, odor discrimination learning and valence encoding.

Both tonic and phasic 10-Hz activation promoted exploration as indexed by increased rearing. Consistent with this outcome, exploratory rearing was previously enhanced with NE or a β-adrenoceptor agonist infused in hippocampus (Flicker and Geyer 1982; Geyer and Masten 1989). Increased immobility, anxiety and aversion, often occurring with 5–10-Hz tonic activation in mice (McCall et al. 2015), were only seen here with 25 Hz (with an output of ~ 15 Hz at the site of the optical stimulation). Possibly optogenetic fiber size in relation to LC extent may support more extensive LC activation in mouse. Spatially extensive recruitment is likely to be stress-encoding. This hypothesis is consistent with the outcome of chemogenetic LC activation (Llorca-Torralba et al. 2019) which seems to invariably induce anxiety and promote place aversions.

The roles of LC–NE in different aspects of olfaction and olfactory learning have been characterized extensively (see reviews Fletcher and Chen 2010; Linster and Escanilla 2019). Here, we compare phasic and tonic LC activation on DOD learning using similar odor pairs in adult rats. Discrimination of similar odors requires NE in the PC (Shakhawat et al. 2015) and the olfactory bulb (OB; Doucette et al. 2007; Mandairon et al. 2008). Increased NE lowers thresholds for odor discrimination (Escanilla et al. 2010) and is associated with higher signal-to-noise ratios (de Almeida et al. 2015). Norepinephrine enhances principle neuron excitability and regulates inhibition in both the OB (Trombley and Shepherd 1992; Hayar et al. 2001; Nai et al. 2009; Nai et al. 2010; Pandipati et al. 2010; Lethbridge et al. 2012) and PC (Gellman and Aghajanian 1993; Brosh et al. 2006; Ghosh et al. 2015) in a dose and receptor subtype-dependent manner, which may contribute to the improvement in signal-to-noise ratio. Norepinephrine enhancement of LTP processes (Yuan et al. 2000; Yuan 2009; Morrison et al. 2013) is also likely to enhance discrimination learning. In adult rats, impaired odor pattern separation is associated with AR blockade (Shakhawat et al. 2015), whereas a short burst of electrical stimulation of the LC sharpens odor representation in the PC (Bouret and Sara 2002). Here we show that phasic, but not tonic LC activation, facilitates odor discrimination learning.

Phasic 10-Hz optogenetic patterns significantly enhanced the rate of acquisition of similar odor discrimination learning. While PC adrenoreceptors are required for similar odor discrimination (see also Shakhawat et al. 2015), it was D1/D5 receptor activation that supported enhancement of acquisition. Increased extracellular DA following LC stimulation to the dorsal hippocampus has been shown to mediate novelty induced learning (Takeuchi et al. 2016) and enhance spatial learning (Kempadoo et al. 2016). In contrast, the source of PC DA in our study was the VTA as shown by prevention of enhancement with VTA lidocaine. Phasic LC activation recruited VTA DA neurons indexed by *cfos*, whereas tonic activation at the same frequency did not enhance acquisition and did not activate VTA DA neurons. Thus, LC output was directed to VTA by the 10-Hz phasic, but not the 10-Hz tonic optogenetic pattern. A recent paper (Soares-Cunha et al. 2019) reported that NAc neurons signal both reward and aversion depending on the optical stimulation patterns. Brief phasic pattern induces reward, whereas prolonged tonic-like stimulation leads to aversion. Interestingly, only the brief phasic stimulation is associated with increased VTA DA tone, similar to what we observed in our study.

Pauses in the phasic pattern are likely to be critical features of the mechanistic effects of phasic patterns. The LC firing pauses may reset target receptor desensitization and have been identified as required for spatial encoding consolidation in slow wave sleep (Swift et al. 2018). Preventing receptor desensitization may enhance both cell recruitment by an input and plasticity during learning. Pauses are also required for DA’s role as a prediction error and teaching signal (Chang et al. 2018).

We also compare the 2 LC activation patterns on odor valence. Tonic LC patterns promote aversions and anxiety in mice (McCall et al. 2015, 2017) and rats (Hirschberg et al. 2017; Llorca-Torralba et al. 2019). A conditioned place aversion and increased anxiety-like behavior have been demonstrated by increasing tonic firing of prefrontal cortex-projecting (Hirschberg et al. 2017) and BLA-projecting LC neurons (McCall et al. 2017; Llorca-Torralba et al. 2019). While phasic LC activation is regarded as learning-promoting, our result suggests that phasic LC activity also carries positive valence.

By prior pairing of an odorant with either 10-Hz phasic or 25-Hz tonic activation, a conditioned odor preference or conditioned odor aversion was acquired respectively. Prior studies suggested aversive place conditioning depended on β-adrenoceptors in the BLA (McCall et al. 2017). Here adrenoceptor involvement was again confirmed for conditioned odor aversion learning. The BLA adrenoceptor activation was also required for conditioned odor preference learning. However, the subsets of basolateral neurons recruited by the phasic and tonic patterns of LC activation were significantly different. Positive valence induced by 10-Hz phasic LC activation stimulated the BLA neurons projecting to NAc, while tonic LC activation at 25 Hz stimulated BLA neurons projecting to CeA. The BLA-NAc pathway has been previously associated with reward, whereas BLA output to CeA is linked to aversive effects (Namburi et al. 2015; Kim et al. 2016). Although LC–BLA engagement in stress and aversive learning has been well documented (McCall et al. 2017; Uematsu et al. 2017), our result provides evidence that phasic activation of the LC leads to positive valence. In the 1970s, phasic electrical stimulation of LC was identified as having positive valence (Ritter and Stein 1973), but this association was criticized as electrical stimulation lacks specificity and a causal relationship was not established (Wise 1978). The present experiments address these deficiencies and reveal a positive valence associated with phasic LC activation. A caveat is that different anatomical portions or different numerical portions of neurons were activated in these experiments. However, it is unlikely that difference in neuron recruitment is a factor given that our rats were randomly assigned to each group and variations in LC activation would be random among groups. This outcome is consistent with the role for LC-hippocampal fiber activation in promoting reward remapping among place cells (Kaufman et al. 2020).

The mechanisms that underlie the differential activations in downstream structures by LC light patterns remain to be determined. The adrenoceptor heterogeneity in the target structures and differential NE release triggered by LC activation patterns may explain the different outcomes. A brain wide MRI study of the changes engendered by chemogenetic LC suggests such activation increases connectivity in salience and amygdala networks that is related to the density of α1 and β1 adrenoceptors (Zerbi et al. 2019). It will be interesting to see how phasic patterns influence such brain connectivity patterns. Other differences in target area receptor-recruitment as a function of activation frequency have also been described. Koga et al. (2020) compared 1 min of 5-Hz or 20-Hz light trains on LC fibers entering anterior cingulate cortex. They found both 5 and 20 Hz enhanced the frequency of presynaptic glutamate release. Although 5 Hz engaged β-adrenoceptor activation, 20-Hz trains further recruited α1-adrenoceptors to increase inward current.

An increase in presynaptic glutamate release frequency is consistent with the predictions of the “glutamate amplifies noradrenergic effects” (GANE) hypothesis (Mather et al. 2016). GANE proposes that LC effects depend on glutamate network activity in target structures. Koga’s outcome supports a presynaptic effect of NE on glutamate release events. Enhancement of local glutamatergic activity will yield varying behavioral outcomes depending on competition among active glutamate circuits. Indeed, all behavioral outcomes with LC activation depend on the nature of activity in the target structures. Glutamate released onto VTA–DA neurons is enhanced by pre-synaptic α1-adrenoceptors (Velasquez-Martinez et al. 2012), which likely occur in our study where phasic LC light facilitates VTA neuron activation during reward-based odor discrimination training. A recent study suggests that LC activation induced DA increase in the dorsal hippocampus facilitates glutamatergic plasticity via pre-synaptic NMDARs (Sonneborn and Greene 2021). Nonetheless, the requirement for a tight pairing of input and LC activation is not necessary for the effects reported here, as well as the hippocampal-dependent learning (Kempadoo et al. 2016; Takeuchi et al. 2016). The LC timing relative to input is critical, however, for attentional effects (Vazey et al. 2018) and spike-timing dependent plasticity (Seol et al. 2007; Huang et al. 2013).

Although there are many variables to consider in explaining differential behavioral effects of phasic and tonic optogentic patterns, the present data suggest 2 possibilities. One is differential downstream recruitment (different structures or different ensembles within the same structure) due to pattern-dependent LC NE release and adrenoceptor heterogeneity in the target structures. Another hypothesis is that different patterns may recruit different subpopulation of LC neurons that have discrete projections. Anatomical heterogeneity within the LC is now thought to support distinct LC behavioral roles (Schwarz and Luo 2015; Schwarz et al. 2015; Uematsu et al. 2015; Chandler et al. 2019). These ensembles are distributed rather broadly through the LC, but may be differentially sensitive to optogenetic patterns.

We suggest that if the different pattern-dependent outcomes illuminated in the present experiments can be integrated with the structural and functional evidence for LC and downstream ensembles (Schwarz and Luo 2015; Uematsu et al. 2015, 2017; Chandler et al. 2019), we will have novel insights into LC operation with broad implications for both basic and clinical brain science.

## Notes

The authors wish to thank Dr Karl Deisseroth' laboratory at Stanford University for support and providing AAVs. Special thanks to Ms. Charu Ramakrishnan. The authors also wish to thank Drs Lynn Nadel and Susan Sara for helpful discussions and comments. *Conflict of Interest:* None declared.

## Funding

This work was supported by Natural Sciences and Engineering Research Council of Canada discovery grants (RGPIN-2018-04401 to Q.Y. and 261384-2008 to X.C.).

## Supplementary Material

Supplementary_figures_tgab026

## References

[ref1] Abercrombie ED , Jacobs BL. 1987. Single-unit response of noradrenergic neurons in the locus coeruleus of freely moving cats. I. Acutely presented stressful and nonstressful stimuli. J Neurosci. 7:2837–2843. 3625275 10.1523/JNEUROSCI.07-09-02837.1987 PMC6569145

[ref2] Ambroggi F , Ishikawa A, Fields HL, Nicola SM. 2008. Basolateral amygdala neurons facilitate reward-seeking behavior by exciting nucleus accumbens neurons. Neuron. 59:648–661. 18760700 10.1016/j.neuron.2008.07.004 PMC2603341

[ref3] Aransay A , Rodriguez-Lopez C, Garcia-Amado M, Clasca F, Prensa L. 2015. Long-range projection neurons of the mouse ventral tegmental area: a single-cell axon tracing analysis. Front Neuroanat. 9:59. 26042000 10.3389/fnana.2015.00059 PMC4436899

[ref4] Aston-Jones G , Bloom FE. 1981. Norepinephrine-containing locus coeruleus neurons in behaving rats exhibit pronounced responses to non-noxious environmental stimuli. J Neurosci. 1:887–900. 7346593 10.1523/JNEUROSCI.01-08-00887.1981 PMC6564231

[ref5] Aston-Jones G , Cohen JD. 2005. An integrative theory of locus coeruleus-norepinephrine function: adaptive gain and optimal performance. Ann Rev Neurosci. 28:403–450. 16022602 10.1146/annurev.neuro.28.061604.135709

[ref6] Aston-Jones G , Rajkowski J, Kubiak P, Alexinsky T. 1994. Locus coeruleus neurons in monkey are selectively activated by attended cues in a vigilance task. J Neurosci. 14:4467–4480. 8027789 10.1523/JNEUROSCI.14-07-04467.1994 PMC6577022

[ref7] Bari A , Xu S, Pignatelli M, Takeuchi D, Feng J, Li Y, Tonegawa S. 2020. Differential attentional control mechanisms by two distinct noradrenergic coeruleo-frontal cortical pathways. Proc Natl Acad Sci U S A. 117:29080–29089. 33139568 10.1073/pnas.2015635117 PMC7682591

[ref8] Bouret S , Sara SJ. 2002. Locus coeruleus activation modulates firing rate and temporal organization of odour-induced single-cell responses in rat piriform cortex. Eur J Neurosci. 16:2371–2382. 12492432 10.1046/j.1460-9568.2002.02413.x

[ref9] Brosh I , Rosenblum K, Barkai E. 2006. Learning-induced reversal of the effect of noradrenalin on the postburst AHP. J Neurophysiol. 96:1728–1733. 16823026 10.1152/jn.00376.2006

[ref10] Carew SJ , Mukherjee B, MacIntyre ITK, Ghosh A, Li S, Kirouac GJ, Harley CW, Yuan Q. 2018. Pheromone-induced odor associative fear learning in rats. Sci Rep. 8:17701. 30532054 10.1038/s41598-018-36023-w PMC6286391

[ref11] Carter ME , Yizhar O, Chikahisa S, Nguyen H, Adamantidis A, Nishino S, Deisseroth K, de Lecea L. 2010. Tuning arousal with optogenetic modulation of locus coeruleus neurons. Nat Neurosci. 13:1526–1533. 21037585 10.1038/nn.2682 PMC3174240

[ref12] Chandler DJ , Jensen P, McCall JG, Pickering AE, Schwarz LA, Totah NK. 2019. Redefining noradrenergic neuromodulation of behavior: impacts of a modular locus Coeruleus architecture. J Neurosci. 39:8239–8249. 31619493 10.1523/JNEUROSCI.1164-19.2019 PMC6794927

[ref13] Chang CY , Gardner MPH, Conroy JC, Whitaker LR, Schoenbaum G. 2018. Brief, but not prolonged, pauses in the firing of midbrain dopamine neurons are sufficient to produce a conditioned inhibitor. J Neurosci. 38:8822–8830. 30181136 10.1523/JNEUROSCI.0144-18.2018 PMC6181314

[ref14] Datiche F , Cattarelli M. 1996. Catecholamine innervation of the piriform cortex: a tracing and immunohistochemical study in the rat. Brain Res. 710:69–78. 8963680 10.1016/0006-8993(95)01279-6

[ref15] de Almeida L , Reiner SJ, Ennis M, Linster C. 2015. Computational modeling suggests distinct, location-specific function of norepinephrine in olfactory bulb and piriform cortex. Front Comput Neurosci. 9:73. 26136678 10.3389/fncom.2015.00073 PMC4468384

[ref16] Devore S , Lee J, Linster C. 2013. Odor preferences shape discrimination learning in rats. Behav Neurosci. 127:498–504. 23895061 10.1037/a0033329 PMC4908962

[ref17] Dong X , Li S, Kirouac GJ. 2017. Collateralization of projections from the paraventricular nucleus of the thalamus to the nucleus accumbens, bed nucleus of the stria terminalis, and central nucleus of the amygdala. Brain Struct Funct. 222:3927–3943. 28528379 10.1007/s00429-017-1445-8

[ref18] Doucette W , Milder J, Restrepo D. 2007. Adrenergic modulation of olfactory bulb circuitry affects odor discrimination. Learn Mem. 14:539–547. 17686948 10.1101/lm.606407 PMC1951793

[ref19] Escanilla O , Arrellanos A, Karnow A, Ennis M, Linster C. 2010. Noradrenergic modulation of behavioral odor detection and discrimination thresholds in the olfactory bulb. Eur J Neurosci. 32:458–468. 20618829 10.1111/j.1460-9568.2010.07297.x

[ref20] Fletcher ML , Chen WR. 2010. Neural correlates of olfactory learning: critical role of centrifugal neuromodulation. Learn Mem. 17:561–570. 20980444 10.1101/lm.941510 PMC2981412

[ref21] Flicker C , Geyer MA. 1982. The hippocampus as a possible site of action for increased locomotion during intracerebral infusions of norepinephrine. Behav Neural Biol. 34:421–426. 7126090 10.1016/s0163-1047(82)91843-x

[ref22] Foote SL , Aston-Jones G, Bloom FE. 1980. Impulse activity of locus coeruleus neurons in awake rats and monkeys is a function of sensory stimulation and arousal. Proc Natl Acad Sci U S A. 77:3033–3037. 6771765 10.1073/pnas.77.5.3033 PMC349541

[ref23] Gellman RL , Aghajanian GK. 1993. Pyramidal cells in piriform cortex receive a convergence of inputs from monoamine activated GABAergic interneurons. Brain Res. 600:63–73. 8422591 10.1016/0006-8993(93)90402-9

[ref24] Geyer MA , Masten VL. 1989. Increases in diversive exploration in rats during hippocampal microinfusions of isoproterenol but not methoxamine. Physiol Behav. 45:213–217. 2543005 10.1016/0031-9384(89)90188-1

[ref25] Ghosh A , Purchase NC, Chen X, Yuan Q. 2015. Norepinephrine modulates pyramidal cell synaptic properties in the anterior piriform cortex of mice: age-dependent effects of beta-adrenoceptors. Front Cell Neurosci. 9:450. 26635530 10.3389/fncel.2015.00450 PMC4652601

[ref26] Hayar A , Heyward PM, Heinbockel T, Shipley MT, Ennis M. 2001. Direct excitation of mitral cells via activation of alpha1-noradrenergic receptors in rat olfactory bulb slices. J Neurophysiol. 86:2173–2182. 11698509 10.1152/jn.2001.86.5.2173

[ref27] Herve-Minvielle A , Sara SJ. 1995. Rapid habituation of auditory responses of locus coeruleus cells in anaesthetized and awake rats. Neuroreport. 6:1363–1368. 7488725 10.1097/00001756-199507100-00001

[ref28] Hirschberg S , Li Y, Randall A, Kremer EJ, Pickering AE. 2017. Functional dichotomy in spinal- vs prefrontal-projecting locus coeruleus modules splits descending noradrenergic analgesia from ascending aversion and anxiety in rats. eLife. 6. 10.7554/eLife.29808 PMC5653237 29027903

[ref29] Huang S , Huganir RL, Kirkwood A. 2013. Adrenergic gating of Hebbian spike-timing-dependent plasticity in cortical interneurons. J Neurosci. 33:13171–13178. 23926270 10.1523/JNEUROSCI.5741-12.2013 PMC3735889

[ref30] Kaufman AM , Geiller T, Losonczy A. 2020. A role for the locus Coeruleus in hippocampal CA1 place cell reorganization during spatial reward learning. Neuron. 105(1018–1026):e1014. 10.1016/j.neuron.2019.12.029 PMC7265133 31980319

[ref31] Kempadoo KA , Mosharov EV, Choi SJ, Sulzer D, Kandel ER. 2016. Dopamine release from the locus coeruleus to the dorsal hippocampus promotes spatial learning and memory. Proc Natl Acad Sci U S A. 113:14835–14840. 27930324 10.1073/pnas.1616515114 PMC5187750

[ref32] Kim J , Pignatelli M, Xu S, Itohara S, Tonegawa S. 2016. Antagonistic negative and positive neurons of the basolateral amygdala. Nat Neurosci. 19:1636–1646. 27749826 10.1038/nn.4414 PMC5493320

[ref33] Koga K , Yamada A, Song Q, Li XH, Chen QY, Liu RH, Ge J, Zhan C, Furue H, Zhuo M, et al. 2020. Ascending noradrenergic excitation from the locus coeruleus to the anterior cingulate cortex. Mol Brain. 13:49. 32216807 10.1186/s13041-020-00586-5 PMC7098117

[ref34] Lechner SM , Curtis AL, Brons R, Valentino RJ. 1997. Locus coeruleus activation by colon distention: role of corticotropin-releasing factor and excitatory amino acids. Brain Res. 756:114–124. 9187321 10.1016/s0006-8993(97)00116-9

[ref35] Lethbridge R , Hou Q, Harley CW, Yuan Q. 2012. Olfactory bulb glomerular NMDA receptors mediate olfactory nerve potentiation and odor preference learning in the neonate rat. PLoS One. 7:e35024. 22496886 10.1371/journal.pone.0035024 PMC3319620

[ref36] Linster C , Escanilla O. 2019. Noradrenergic effects on olfactory perception and learning. Brain Res. 1709:33–38. 29574010 10.1016/j.brainres.2018.03.021 PMC6150838

[ref37] Llorca-Torralba M , Suarez-Pereira I, Bravo L, Camarena-Delgado C, Garcia-Partida JA, Mico JA, Berrocoso E. 2019. Chemogenetic silencing of the locus Coeruleus-basolateral amygdala pathway abolishes pain-induced anxiety and enhanced aversive learning in rats. Biol Psychiatry. 85:1021–1035. 30987747 10.1016/j.biopsych.2019.02.018

[ref38] Mandairon N , Peace S, Karnow A, Kim J, Ennis M, Linster C. 2008. Noradrenergic modulation in the olfactory bulb influences spontaneous and reward-motivated discrimination, but not the formation of habituation memory. Eur J Neurosci. 27:1210–1219. 18364038 10.1111/j.1460-9568.2008.06101.x

[ref39] Mather M , Clewett D, Sakaki M, Harley CW. 2016. Norepinephrine ignites local hotspots of neuronal excitation: how arousal amplifies selectivity in perception and memory. Behav Brain Sci. 39:e200. 26126507 10.1017/S0140525X15000667 PMC5830137

[ref40] McCall JG , Al-Hasani R, Siuda ER, Hong DY, Norris AJ, Ford CP, Bruchas MR. 2015. CRH engagement of the locus coeruleus noradrenergic system mediates stress-induced anxiety. Neuron. 87:605–620. 26212712 10.1016/j.neuron.2015.07.002 PMC4529361

[ref41] McCall JG , Siuda ER, Bhatti DL, Lawson LA, McElligott ZA, Stuber GD, Bruchas MR. 2017. Locus coeruleus to basolateral amygdala noradrenergic projections promote anxiety-like behavior. eLife. 6. 10.7554/eLife.18247 PMC5550275 28708061

[ref42] Morrison GL , Fontaine CJ, Harley CW, Yuan Q. 2013. A role for the anterior piriform cortex in early odor preference learning: evidence for multiple olfactory learning structures in the rat pup. J Neurophysiol. 110:141–152. 23576704 10.1152/jn.00072.2013 PMC4073989

[ref43] Nai Q , Dong HW, Hayar A, Linster C, Ennis M. 2009. Noradrenergic regulation of GABAergic inhibition of main olfactory bulb mitral cells varies as a function of concentration and receptor subtype. J Neurophysiol. 101:2472–2484. 19279145 10.1152/jn.91187.2008 PMC2681435

[ref44] Nai Q , Dong HW, Linster C, Ennis M. 2010. Activation of alpha1 and alpha 2 noradrenergic receptors exert opposing effects on excitability of main olfactory bulb granule cells. Neuroscience. 169:882–892. 20466037 10.1016/j.neuroscience.2010.05.010 PMC2904409

[ref45] Nakamura S , Kimura F, Sakaguchi T. 1987. Postnatal development of electrical activity in the locus ceruleus. J Neurophys. 58:510–524. 10.1152/jn.1987.58.3.510 3655880

[ref46] Namburi P , Al-Hasani R, Calhoon GG, Bruchas MR, Tye KM. 2016. Architectural representation of valence in the limbic system. Neuropsychopharmacology. 41:1697–1715. 26647973 10.1038/npp.2015.358 PMC4869057

[ref47] Namburi P , Beyeler A, Yorozu S, Calhoon GG, Halbert SA, Wichmann R, Holden SS, Mertens KL, Anahtar M, Felix-Ortiz AC, et al. 2015. A circuit mechanism for differentiating positive and negative associations. Nature. 520:675–678. 25925480 10.1038/nature14366 PMC4418228

[ref48] Nunes EJ , Bitner L, Hughley SM, Small KM, Walton SN, Rupprecht LE, Addy NA. 2019. Cholinergic receptor blockade in the VTA attenuates cue-induced cocaine-seeking and reverses the anxiogenic effects of forced abstinence. Neuroscience. 413:252–263. 31271832 10.1016/j.neuroscience.2019.06.028 PMC6661179

[ref49] O'Neill PK , Gore F, Salzman CD. 2018. Basolateral amygdala circuitry in positive and negative valence. Curr Opin Neurobiol. 49:175–183. 29525574 10.1016/j.conb.2018.02.012 PMC6138049

[ref50] Pandipati S , Gire DH, Schoppa NE. 2010. Adrenergic receptor-mediated disinhibition of mitral cells triggers long-term enhancement of synchronized oscillations in the olfactory bulb. J Neurophysiol. 104:665–674. 20538781 10.1152/jn.00328.2010 PMC2934928

[ref51] Quinlan MAL , Strong VM, Skinner DM, Martin GM, Harley CW, Walling SG. 2018. Locus coeruleus optogenetic light activation induces long-term potentiation of perforant path population spike amplitude in rat dentate gyrus. Front Syst Neurosci. 12:67. 30687027 10.3389/fnsys.2018.00067 PMC6333706

[ref52] Rajkowski J , Kubiak P, Aston-Jones G. 1994. Locus coeruleus activity in monkey: phasic and tonic changes are associated with altered vigilance. Brain Res Bull. 35:607–616. 7859118 10.1016/0361-9230(94)90175-9

[ref53] Ritter S , Stein L. 1973. Self-stimulation of noradrenergic cell group (A6) in locus coeruleus of rats. J Comp Physiol Psychol. 85:443–452. 4586874 10.1037/h0035289

[ref54] Rodenkirch C , Liu Y, Schriver BJ, Wang Q. 2019. Locus coeruleus activation enhances thalamic feature selectivity via norepinephrine regulation of intrathalamic circuit dynamics. Nat Neurosci. 22:120–133. 30559472 10.1038/s41593-018-0283-1 PMC6301066

[ref55] Rodriguez-Manzo G , Canseco-Alba A. 2017. A new role for GABAergic transmission in the control of male rat sexual behavior expression. Behav Brain Res. 320:21–29. 27908749 10.1016/j.bbr.2016.11.041

[ref56] Sara SJ , Segal M. 1991. Plasticity of sensory responses of locus coeruleus neurons in the behaving rat: implications for cognition. Progr Brain Res. 88:571–585. 10.1016/s0079-6123(08)63835-2 1813935

[ref57] Schwarz LA , Luo L. 2015. Organization of the locus coeruleus-norepinephrine system. Curr Biol. 25:R1051–R1056. 26528750 10.1016/j.cub.2015.09.039

[ref58] Schwarz LA , Miyamichi K, Gao XJ, Beier KT, Weissbourd B, DeLoach KE, Ren J, Ibanes S, Malenka RC, Kremer EJ, et al. 2015. Viral-genetic tracing of the input-output organization of a central noradrenaline circuit. Nature. 524:88–92. 26131933 10.1038/nature14600 PMC4587569

[ref59] Seol GH , Ziburkus J, Huang S, Song L, Kim IT, Takamiya K, Huganir RL, Lee HK, Kirkwood A. 2007. Neuromodulators control the polarity of spike-timing-dependent synaptic plasticity. Neuron. 55:919–929. 17880895 10.1016/j.neuron.2007.08.013 PMC2756178

[ref60] Shakhawat AM , Gheidi A, MacIntyre IT, Walsh ML, Harley CW, Yuan Q. 2015. Arc-expressing neuronal ensembles supporting pattern separation require adrenergic activity in anterior piriform cortex: an exploration of neural constraints on learning. J Neurosci. 35:14070–14075. 26468206 10.1523/JNEUROSCI.2690-15.2015 PMC6608176

[ref61] Shakhawat AM , Harley CW, Yuan Q. 2014. Arc visualization of odor objects reveals experience-dependent ensemble sharpening, separation, and merging in anterior piriform cortex in adult rat. J Neurosci. 34:10206–10210. 25080582 10.1523/JNEUROSCI.1942-14.2014 PMC6608274

[ref62] Sonneborn A , Greene RW. 2021. Norepinephrine transporter antagonism prevents dopamine-dependent synaptic plasticity in the mouse dorsal hippocampus. Neurosci Lett. 740:135450. 33127445 10.1016/j.neulet.2020.135450 PMC7725138

[ref63] Swift KM , Gross BA, Frazer MA, Bauer DS, Clark KJD, Vazey EM, Aston-Jones G, Li Y, Pickering AE, Sara SJ, et al. 2018. Abnormal locus coeruleus sleep activity alters sleep signatures of memory consolidation and impairs place cell stability and spatial memory. Curr Biol. 28(3599–3609):e3594. 10.1016/j.cub.2018.09.054 PMC7556718 30393040

[ref64] Takeuchi T , Duszkiewicz AJ, Sonneborn A, Spooner PA, Yamasaki M, Watanabe M, Smith CC, Fernandez G, Deisseroth K, Greene RW, et al. 2016. Locus coeruleus and dopaminergic consolidation of everyday memory. Nature. 537:357–362. 27602521 10.1038/nature19325 PMC5161591

[ref65] Trombley PQ , Shepherd GM. 1992. Noradrenergic inhibition of synaptic transmission between mitral and granule cells in mammalian olfactory bulb cultures. J Neurosci. 12:3985–3991. 1328562 10.1523/JNEUROSCI.12-10-03985.1992 PMC6575954

[ref66] Uematsu A , Tan BZ, Johansen JP. 2015. Projection specificity in heterogeneous locus coeruleus cell populations: implications for learning and memory. Learn Mem. 22:444–451. 26330494 10.1101/lm.037283.114 PMC4561410

[ref67] Uematsu A , Tan BZ, Ycu EA, Cuevas JS, Koivumaa J, Junyent F, Kremer EJ, Witten IB, Deisseroth K, Johansen JP. 2017. Modular organization of the brainstem noradrenaline system coordinates opposing learning states. Nat Neurosci. 20:1602–1611. 28920933 10.1038/nn.4642

[ref68] Vazey EM , Moorman DE, Aston-Jones G. 2018. Phasic locus coeruleus activity regulates cortical encoding of salience information. Proc Natl Acad Sci U S A. 115:E9439–E9448. 30232259 10.1073/pnas.1803716115 PMC6176602

[ref69] Velasquez-Martinez MC , Vazquez-Torres R, Jimenez-Rivera CA. 2012. Activation of alpha1-adrenoceptors enhances glutamate release onto ventral tegmental area dopamine cells. Neuroscience. 216:18–30. 22542873 10.1016/j.neuroscience.2012.03.056 PMC3809080

[ref70] Wise RA . 1978. Catecholamine theories of reward: a critical review. Brain Res. 152:215–247. 354753 10.1016/0006-8993(78)90253-6

[ref71] Yuan Q . 2009. Theta bursts in the olfactory nerve paired with beta-adrenoceptor activation induce calcium elevation in mitral cells: a mechanism for odor preference learning in the neonate rat. Learn Mem. 16:676–681. 19858361 10.1101/lm.1569309 PMC2775516

[ref72] Yuan Q , Harley CW, Bruce JC, Darby-King A, McLean JH. 2000. Isoproterenol increases CREB phosphorylation and olfactory nerve-evoked potentials in normal and 5-HT-depleted olfactory bulbs in rat pups only at doses that produce odor preference learning. Learn Mem. 7:413–421. 11112800 10.1101/lm.35900 PMC311343

[ref73] Yun IA , Wakabayashi KT, Fields HL, Nicola SM. 2004. The ventral tegmental area is required for the behavioral and nucleus accumbens neuronal firing responses to incentive cues. J Neurosci. 24:2923–2933. 15044531 10.1523/JNEUROSCI.5282-03.2004 PMC6729854

[ref74] Zerbi V , Floriou-Servou A, Markicevic M, Vermeiren Y, Sturman O, Privitera M, von Ziegler L, Ferrari KD, Weber B, De Deyn PP, Wenderoth N, Bohacek J. 2019. Rapid reconfiguration of the functional connectome after chemogenetic locus Coeruleus activation. Neuron 103:702–718 e705. 10.1016/j.neuron.2019.05.034 31227310

