# Supplementary material for: Locus Coeruleus Activation Patterns Differentially Modulate Odor Discrimination Learning and Odor Valence in Rats
Source: Cereb Cortex Commun. 2021 Apr 5;2(2):tgab026. doi: 10.1093/texcom/tgab026 (PMC8152946; doi:10.1093/texcom/tgab026)
Supplement: Supplementary_figures_tgab026 [file supplementary_figures_tgab026.pdf]

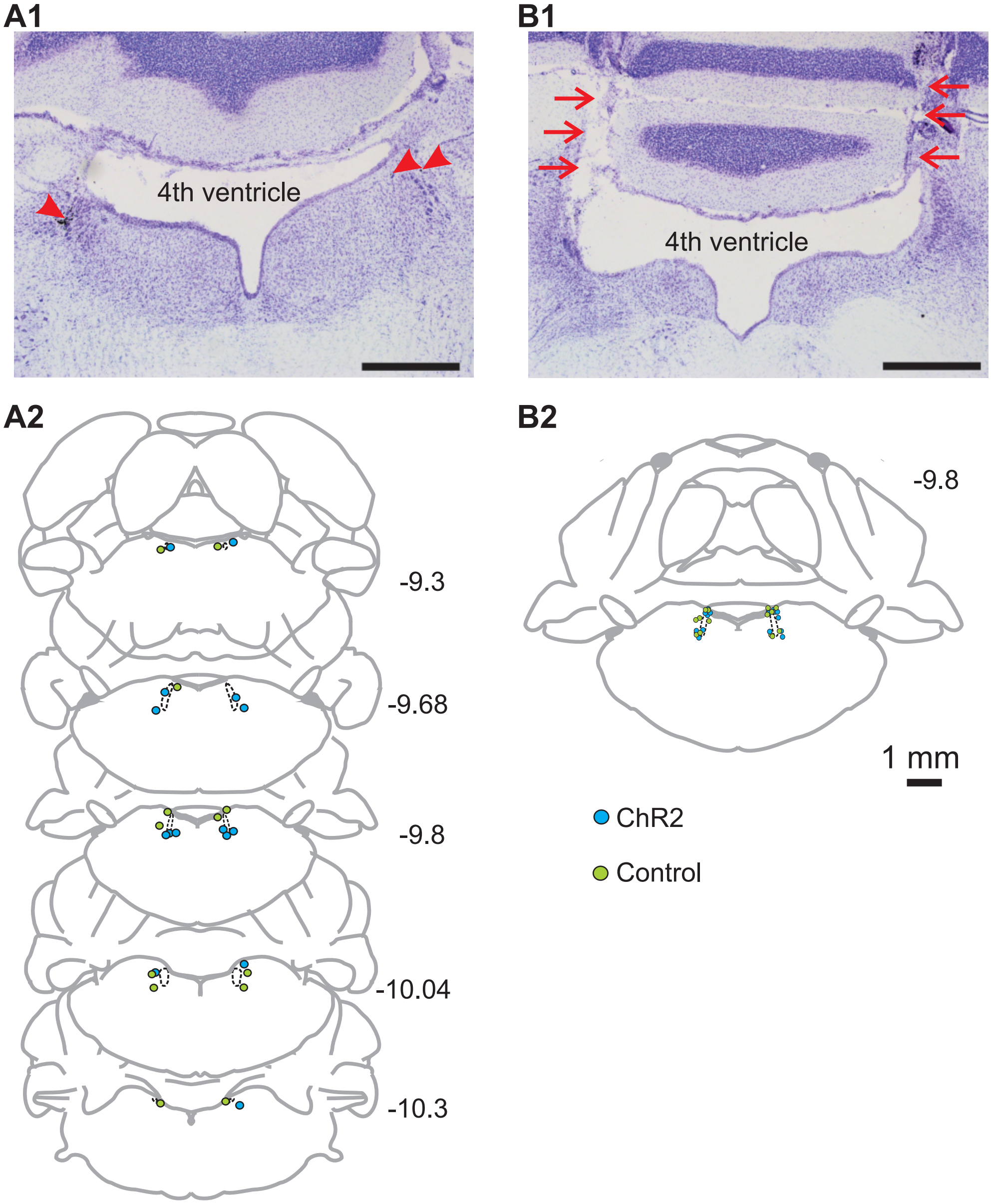


**Supplementary Figure 1. LC targeting summary**
**A1.** An example of blue beads infused into the LC. Arrow heads indicate visible blue beads. **A2.** Schematics showing the infusion sites from ChR2 and control rats at different coronal levels (ChR2: n = 8; Control: n = 8). **B1.** An example of cannula tracks (indicated by red arrows bilaterally). **B2.** Schematics showing the cannular sites from ChR2 and control rats at -9.8 level (ChR2: n = 8; Control: n = 8). Scale bars for A1 and B1, 500 μm.

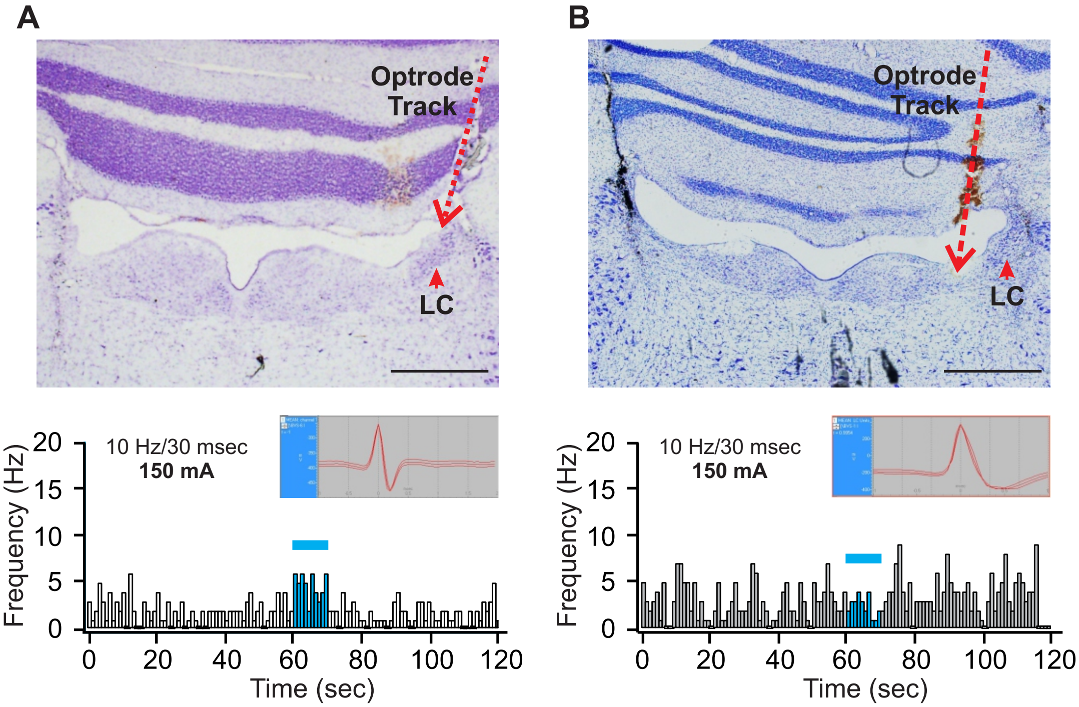


**Supplementary Figure 2. Light stimulation induces increased spiking in LC neurons.**
**A.** An example of correct LC targeting of an optrode recording and associated cell response. Blue bar indicates period of light stimulation. The inset shows the spiking waveform of the cell.
**B.** A mis-targeted cell did not exhibit light-induced spiking increase. Scale bars: 500 µm.

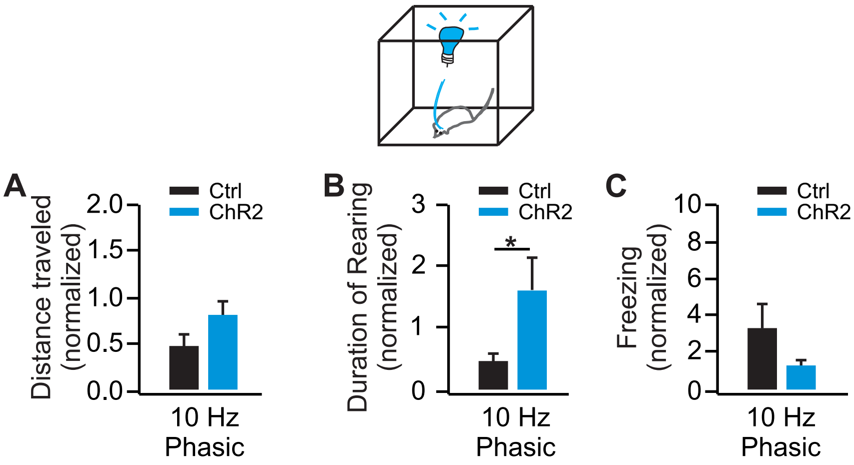


**Supplementary Figure 3. Ten-Hz brief phasic LC activation increases exploration in rats**
**A.** Distance traveled in the open field. **B.** Duration of rearing in the open field. **C.** Percentage freezing in the open field (ChR2: n = 7; Control: n = 8). *$p < 0.05$.

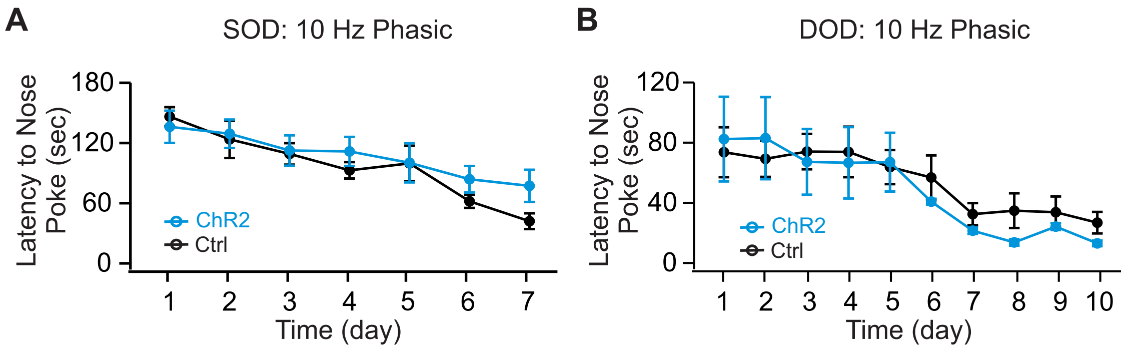


**Supplementary Figure 4. Latency to nose poke during SOD (A) and DOD (B) associated with 10-Hz phasic LC stimulation**

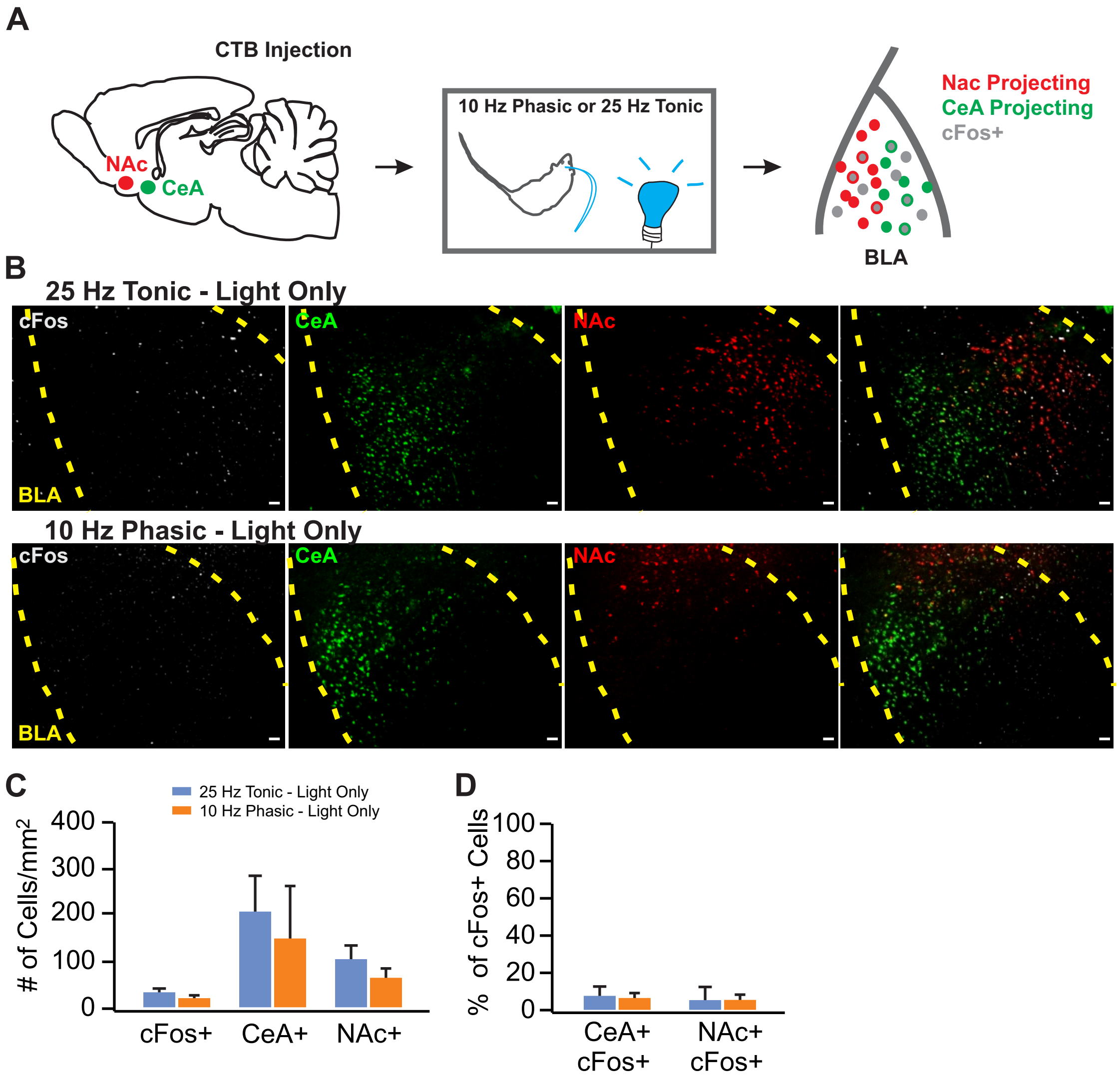


**Supplementary Figure 5. Ten-Hz phasic and 25-Hz tonic LC activations do not induce distinct cFos activation patterns in the BLA in the absence of an odor**

**A.** Schematic of measuring cFos activation in the BLA with CTB labeling NAc and CeA projecting neurons. **B.** Examples images of cFos, CTB-488 (labeling CeA projecting neurons) and CTB-594 (labeling NAc projecting neurons) in the BLA with 25-Hz light only (upper panels), and 10-Hz phasic light only (lower panels). Scale bars, 50 µm. **C.** Total $cFos^+$, $CeA^+$ and $NAc^+$ cells activated by tonic and phasic lights). **D.** Percentage $CeA^+/cFos^+$ and $NAc^+/cFos^+$ cells over total $cFos^+$ population.

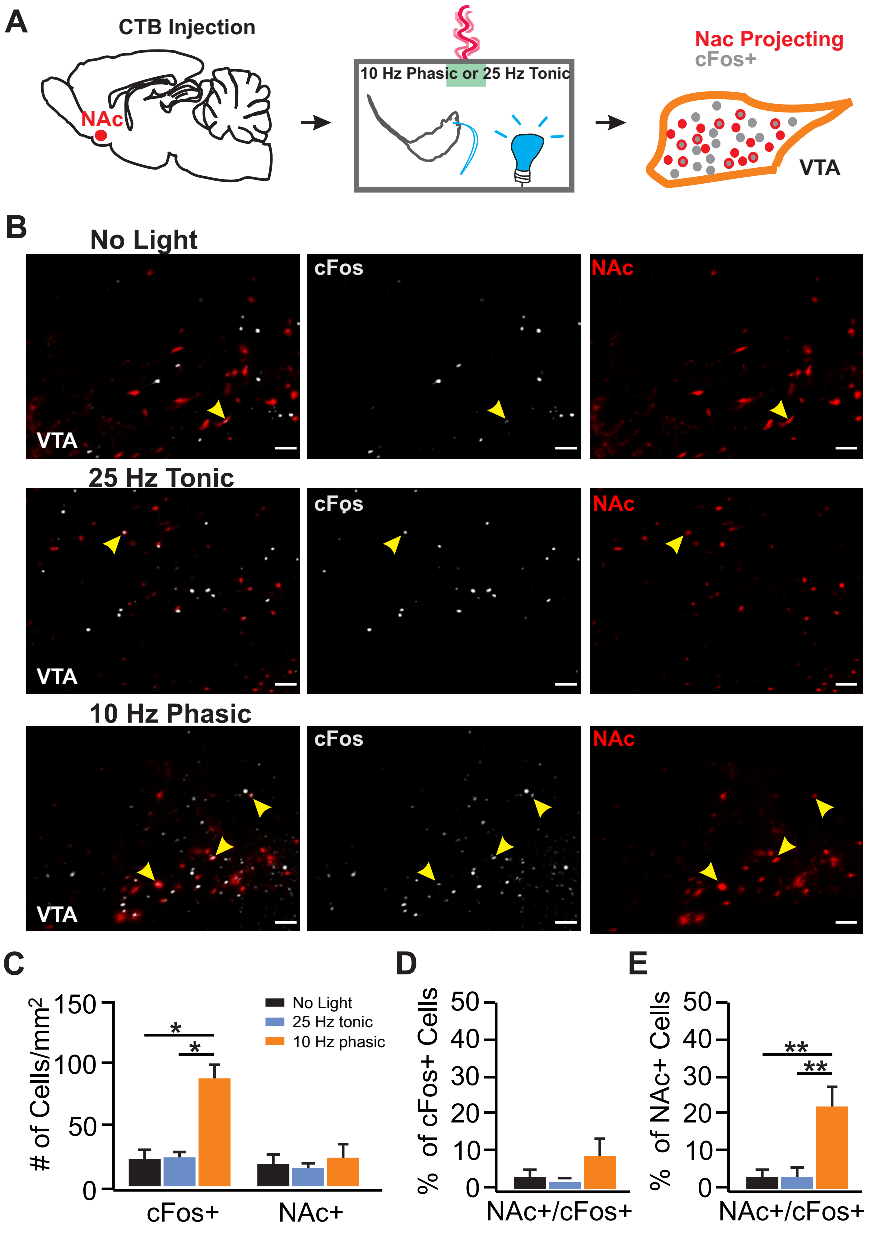


**Supplementary Figure 6. Ten-Hz phasic, but not 25-Hz tonic, LC activation engages NAc projecting neurons in the VTA**

**A.** Schematic of measuring cFos activation in the VTA with CTB labeling NAc-projecting neurons. **B.** Examples images of cFos and CTB-594 (labeling NAc-projecting neurons) in no-light control (upper panels), activated by 25-Hz tonic (middle panels) and 10-Hz brief phasic light (300 msec every 2 sec; lower panels). Scale bars, 50 µm. **C.** Total $cFos^{+}$ and $NAc^{+}$ cells activated in different groups (n (control/tonic/phasic) = 4/4/3). **D.** Percentage of $cFos^{+}$ cells that are $NAc^{+}$. **E.** Percentage of $NAc^{+}$ cells that are $cFos^{+}$. *$p < 0.05$. **$p < 0.01$
